# Unraveling the mechanisms of surround suppression in early visual processing

**DOI:** 10.1371/journal.pcbi.1008916

**Published:** 2021-04-22

**Authors:** Yao Li, Lai-Sang Young

**Affiliations:** 1 Department of Mathematics and Statistics, University of Massachusetts Amherst, Amherst, Massachusetts, United States of America; 2 Courant Institute of Mathematical Sciences, New York University, New York, New York, United States of America; 3 Institute for Advanced Study, Princeton, New Jersey, United States of America; Shanghai Jiao Tong University, CHINA

## Abstract

This paper uses mathematical modeling to study the mechanisms of surround suppression in the primate visual cortex. We present a large-scale neural circuit model consisting of three interconnected components: LGN and two input layers (Layer 4Ca and Layer 6) of the primary visual cortex V1, covering several hundred hypercolumns. Anatomical structures are incorporated and physiological parameters from realistic modeling work are used. The remaining parameters are chosen to produce model outputs that emulate experimentally observed size-tuning curves. Our two main results are: (i) we discovered the character of the long-range connections in Layer 6 responsible for surround effects in the input layers; and (ii) we showed that a net-inhibitory feedback, i.e., feedback that excites I-cells more than E-cells, from Layer 6 to Layer 4 is conducive to producing surround properties consistent with experimental data. These results are obtained through parameter selection and model analysis. The effects of nonlinear recurrent excitation and inhibition are also discussed. A feature that distinguishes our model from previous modeling work on surround suppression is that we have tried to reproduce realistic lengthscales that are crucial for quantitative comparison with data. Due to its size and the large number of unknown parameters, the model is computationally challenging. We demonstrate a strategy that involves first locating baseline values for relevant parameters using a linear model, followed by the introduction of nonlinearities where needed. We find such a methodology effective, and propose it as a possibility in the modeling of complex biological systems.

## Author summary

The visual cortex is a part of the cortex that processes visual signal. The visual signal from the retina is relayed through the lateral geniculate nucleus (LGN), and enters the primary visual cortex directly. The primary visual cortex consists of many layers. A phenomenon called surround suppression, which means a reduction of neuronal activities in response to stimuli of increasing size, is well-observed in all layers of the primate primary visual cortex. In this paper, we built a large scale mathematical model consisting of the LGN input and tens of thousands of neuron groups in Layer 4 and Layer 6 of the primary visual cortex to study the mechanism of surround suppression. This model is constrained by both anatomical structures and realistic physiological parameters. Remaining unknown parameters are reverse-engineered from experimental data. We showed how the character of the surround suppression depends on the long-range connections in the feedback layer, as well as the feedback current from Layer 6 to Layer 4.

## 1 Introduction

The goal of this paper is to discover mechanisms behind certain phenomena in visual neuroscience via mathematical modeling and simulations. The neural phenomena in question have to do with surround modulation, a version of which can be described as follows: In the mammalian cortex, the first stage of visual signal processing takes place in the primary visual cortex, also known as V1. Neurons in V1 receive information directly from the retina relayed through the lateral geniculate nucleus (LGN). V1 cells are known to have very small apertures, meaning the part of the visual field about which information is relayed directly to each cell is very small, no more than a fraction of a degree. This part of the visual field is known as the cell’s classical receptive field (CRF). Interestingly, properties of the stimulus well outside of a cell’s CRF can substantially influence the cell’s response. Specifically, cells in V1 respond vigorously to certain small stimuli centered at their CRF but their firing rates are attenuated when the eye is presented with a larger size stimulus of the same kind. This phenomenon is known as *surround suppression*: stimulating the cortical region around a cell can suppress the cell’s response. Surround suppression is one of the most basic and most relevant visual phenomena in the sense that it affects almost everything we see, and it has been well documented in the experimental literature for quite some time, yet the mechanisms for it have yet to be understood. We refer the reader to the review article [1]. In this paper we would like to contribute to solving a small piece of this very complex puzzle.

In general, a neuron’s dynamics are driven by feedforward inputs, and modulated by feedback and lateral interactions with other neurons. Now V1 is composed of several layers, and it is likely that the mechanisms for surround suppression in the different layers are not identical because the layers have different anatomical structures and receive information from different sources; again see [1]. We will work with input layers of the magno-pathway: input layers because one has better control of the signal when it first enters the primary visual cortex and structures of the input layers are simpler; the magno-pathway is selected because surround effects are more pronounced in input layers of the magno (as opposed to the parvo) pathway.

**Fig 1:**
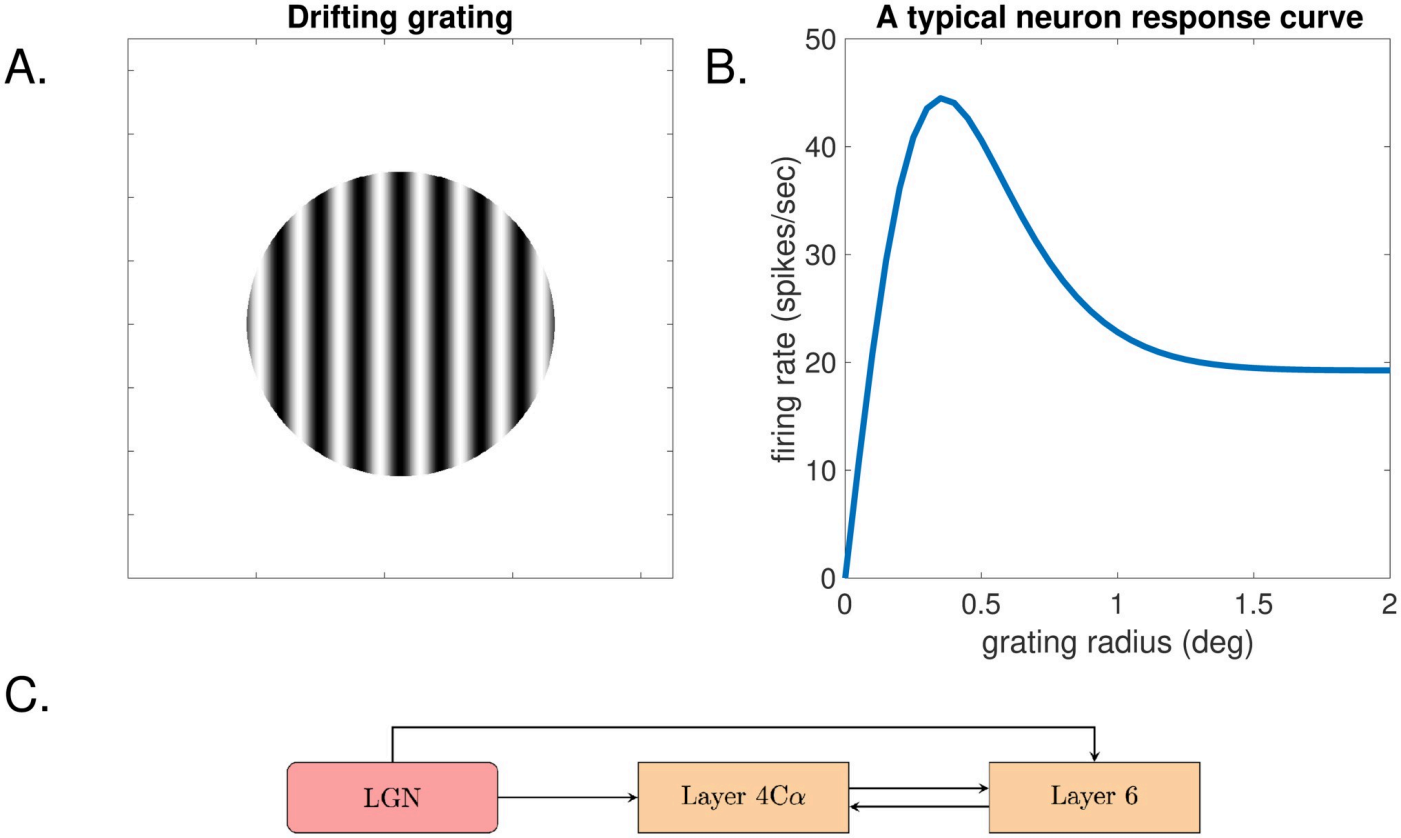
The phenomenon of surround suppression. A. Drifting grating. B. Response of a typical neuron in response to drifting gratings of radius *R*, plotted as function of *R*. C. A diagram of the LGN-L4-L6 circuit.

Having localized the biological problem to make it more accessible, we now discuss the mathematical problem before us. We work with a 3-component circuit (see Fig 1C) the rough anatomical structures of which are known. Following these structures, we set up a large network the nodes of which are small groups of Excitatory or Inhibitory neurons. The two big unknowns for us are (i) connection probabilities, i.e., the probability of a group being connected to another group, and (ii) synaptic coupling weights, e.g., the extent to which the arousal of a group of Excitatory neurons excites the groups postsynaptic to it. These quantities for real cortex are unknown and are not easily measured in the laboratory—yet they are essential for unraveling cortical mechanisms. Specifically, experimentalists have measured the firing rate responses of neurons at the “center” to drifting gratings (Fig 1A) of increasing radii, producing *size-tuning curves* of the kind shown in Fig 1B. These curves will serve as targets for the model to emulate. In general terms, we have in our hands a large and complex dynamical system with a number of unknown parameters. Our primary challenges are to reverse-engineer these parameters from the system’s responses to external stimuli, and then to use the information gained to explain the mechanisms that produce these responses. A secondary aim of this paper is to develop techniques for solving “inverse problems” of this kind associated with large-scale neuronal models.

Needless to say, we are not the first theorists to work on the problem of surround suppression. Very general theories of canonical computation of cortical circuits have been proposed by neuroscientists, including the normalization ideas of [2], the ideas of inhibition-stabilized networks [3] and of stabilized supralinear networks [4]. Abstracting from experimental observations of how neurons respond to multiple inputs of different strengths, the authors of these papers proposed very general phenomenological models to explain a wide range of neural phenomena including surround suppression. They offered broad principles of neuronal behaviors, but the models proposed are not constrained by data. There are other descriptive models. We mention in particular [5], which proposed that suppression comes from a group of neurons external to the population in question, an intriguing idea the biological basis of which remains to be confirmed.

Overall, there are surprisingly few proposed theories for a phenomenon as important as surround suppression that are directly tied to experimental data. This is what drew us to the problem. We have focused on a very specific part of the visual cortex, a region that is relatively simple and rich in data, and have built as much of the relevant neuroanatomy and neurophysiology into the model as we could; we have also borrowed from earlier models that are biologically constrained, e.g. [6]. In trying to solve the problem we found that crucial data were missing, information that can easily be obtained from electrophysiology. At the same time, we have–via a large-scale computational model–come to conclusions that resonate to some degree with the ideas in the phenomenological models discussed in the last paragraph. We will follow up on these points in **Discussion**.

This paper is organized as follows. In **Results**, Section 2.1 contains some neuroscience background on the anatomy of V1 and the phenomenon of surround suppression. Section 2.2 describes the general layout of the model, including LGN input, connectivity, coupling weight, and key parameters. Target model outputs and computational strategies are discussed in Section 2.3. Results for the linear model are presented in Section 2.4. Section 2.5 introduces a nonlinearity to the model and discusses its effect. Section summaries are provided at the end of Sections 2.2, 2.4, and 2.5, and recapitulated in **Discussion**, which contains also discussions of related works. More detailed information on the model is included in **Methods**, and full mathematical details of models and parameters are given in S1 Text.

## 2 Results

### 2.1 Neuroscience background

This paper seeks to provide a mechanistic explanation for surround suppression, one of the most fundamental and most intriguing phenomena in visual neuroscience. In Sect. 2.1.1 we describe the phenomenon, and in Sect. 2.1.2, we fill in some of the relevant neurobiological background for the benefit of readers not familiar with the subject.

#### 2.1.1 What is surround suppression?

Surround suppression is a visual phenomenon—more accurately a body of visual phenomena—that play important roles in shaping our perception of the world around us [1]. Roughly speaking, it refers to the fact that a neuron’s sensitivity to a stimulus is modulated by what lies outside of the neuron’s classical receptive field. In this paper, we will focus on one of the most basic forms of surround suppression, namely that neurons respond more vigorously to stimuli that are smaller and centered at their receptive fields, and less vigorously to stimuli of the same kind but of a larger extent. Counter-intuitive as it may seem, this phenomenon has been observed and clearly documented in experiments, though the neural mechanisms behind it are far from understood.

We are primarily interested in primates, though surround suppression is widely observed across species in many mammals. The part of the cerebral cortex that processes visual information is called the visual cortex, and the first stage of processing takes place in the *primary visual cortex*, or V1. Leaving details to the next subsection, we mention here two facts about neurons in V1 that will enable us to describe the phenomenon of interest. One is that neurons in V1 have very small *receptive fields*, about a quarter the size of the moon when we gaze at objects directly in front of us. The receptive field of a neuron is the region of visual space about which the neuron receives direct feedforward information from the retina. The second relevant fact is that neurons in V1 are *orientation selective*, meaning each neuron has a preferred orientation: it is excited by the presence of an edge in its receptive field in that orientation. Suppose a neuron prefers vertical edges. Then its firing rate is high when there is a vertical edge in its receptive field, lower when the edge makes a 30° angle with the vertical, and a horizontal edge will not elicit much activity beyond background levels.

Because of the orientation selectivity of neurons in V1, and the fact that V1 neurons are primarily detectors of changes in luminance, neuroscientists have often used *drifting gratings* (Fig 1A) as visual stimuli to target neurons that prefer particular orientations. The most basic form of surround suppression can now be described as follows: Consider the response of a neuron to a family of drifting gratings of different radii *R*, all centered at the neuron’s receptive field and with the bars in the gratings aligned with the neuron’s preferred orientation. The neuron’s responses to the different gratings, measured in number of spikes per second, are then recorded and plotted against the radius *R* of the grating. Typical experimental results are as shown in Fig 1B: As *R* increases from 0, the firing rate increases; it peaks at a certain radius, and then starts to decrease, leveling off eventually at a fairly large radius. Interestingly, the peaking and subsequent decrease in firing rate occur at values of *R* larger than the receptive field of the neuron. That is, the neuron responds to the part of the grating outside of its receptive field, and the larger the grating, the more attenuated its response. Explaining the neural mechanism behind this is the challenge before us.

#### 2.1.2 Some relevant neuroanatomy

This paper studies surround suppression in the input layers to V1. Our primary focus is Layer 4*Cα*, but because Layer 4*Cα* interacts with other layers, it is necessary to consider, at a minimum, the circuit involving LGN, Layer 4*Cα* and Layer 6 of V1 (Fig 1C), and that is what we will study in this paper. We will abbreviate this as the LGN-L4-L6 circuit. While the reader can take our model as described in next subsection as starting point, we provide below some basic information on the actual biology so the reader can get a sense of the origin of the model.

Visual signals pass from the retina to two small structures in the thalamus called the lateral geniculate nuclei (LGN) and from there directly to the primary visual cortex, V1. They travel along two separate pathways, the magnocellular pathway (which has high sensitivity to contrast) and the parvocellular pathway (used for perception of fine details and color). LGN has several layers, some magno some parvo; we are interested in one of the magno layers. The involvement of LGN is essential because there is evidence that part of the surround suppression in cortex is inherited from LGN, and ultimately probably from the retina [7].

The primary visual cortex, V1, which is roughly speaking a 2D sheet of neural tissues, is also is made up of a number of layers. But unlike LGN, there is a large number of cortico-cortical connections among neurons within each layer, as well as some inter-laminar connections [8, 9]. Layer 4C*α*, which receives direct input from LGN, is the input layer to V1 in the magno-pathway. The only other layer to which Layer 4C*α* projects is Layer 6, which also receives input from LGN and which provides feedback to 4C*α* as indicated in Fig 1C. This unfortunately is not a closed circuit: Layer 6 has complicated internal structures as well as interactions with a number of other layers of V1, something we will neglect in the present study.

One of the most basic facts about how the retina, LGN and visual cortex are related is the existence of *retinotopic maps*. When our eyes are open, there is a 2D map of our visual field on the retina. It turns out that the projections from retina to LGN, from LGN to V1 (and on to higher visual areas) confer in each region a “map”, roughly speaking a point-to-point association between the retina and the 2D surface of V1. Small groups of nearby neurons are associated to roughly the same spatial location, i.e., they share the same receptive fields. Such groups have the structure of columns transversal to the layered structure of V1; one sometimes calls them *hypercolumns*. Each hypercolumn is further subdivided into several smaller columns consisting of neurons having the same orientation preferences, and orientation domains are known to be organized in pinwheel formations around certain singularity points [10].

As to the internal connections within each layer, the different layers of V1 have somewhat different network topologies that may affect their surround properties and mechanisms. All have local circuits consisting of on the order of 1000 neurons, about 75% Excitatory (E) and the rest Inhibitory (I). Pairs of nearby E-neurons have about a 15% chance of being connected, their connection probabilities falling off with distance. All other connections, i.e. between E and I and between I and I, are much denser. Network connectivity in Layer 4C*α* is one of the simplest: it consists primarily of local circuits. Layer 6, on the other hand, has longer-range connections between groups of neurons with like orientation preferences. Detailed information on connectivity within primate Layer 6 is not available, and that is one of the unknowns we will have to confront when we try to understand surround suppression.

For more information, we refer the reader to standard neuroscience texts.

### 2.2 Model description

For our study of surround suppression in the input layers of the magnocellular pathway of the macaque V1 cortex, we use a three-tier model the components of which are

(i) a sheet of cells in the lateral geniculate nucleus (LGN),
(ii) a region of layer 4C*α* (abbreviated as L4 in the rest of this paper) and
(iii) a region of layer 6 (abbreviated as L6).

The cortical layers cover a total of ∼300 hypercolumns, to be thought of as centered at 5 − 10° eccentricity. The model is coarse-grained: we do not model the dynamics of individual neurons, bunching together instead small groups of nearby E and I cells and tracking their outputs. These small groups of neurons interact with one another as they do in real cortex. Spatial extents of projections are modeled carefully, as length scales matter in surround suppression. We present in this section a model description that should be adequate for understanding the results in Sections 2.3 and 2.4. Details of the model including the exact parameters used are given in **Methods**.

#### 2.2.1 General model layout

**Fig 2:**
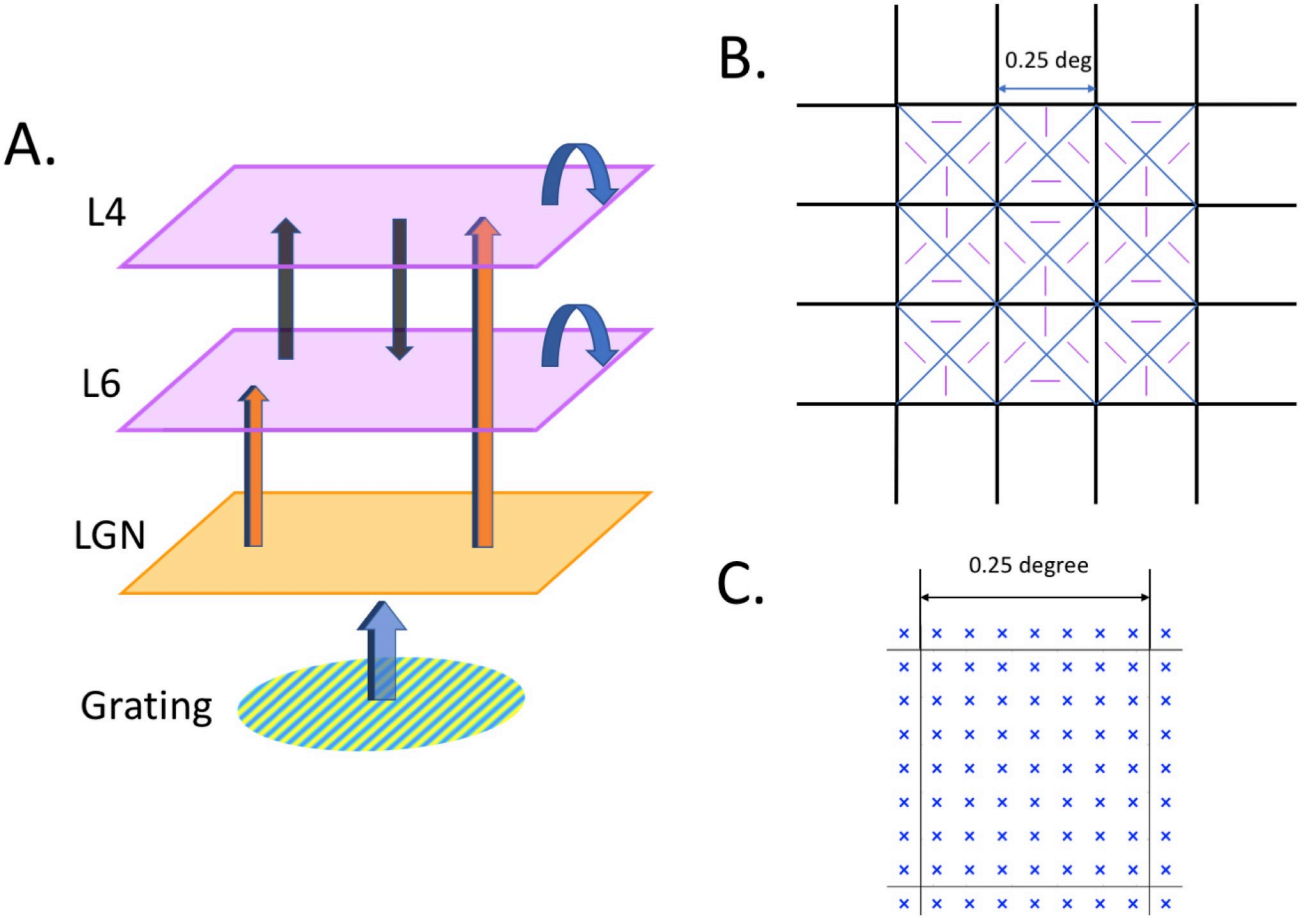
A. The three components of the model. Visual signals are transmitted to LGN and then projected to L4 and L6. Neurons in L4 and L6 communicate with neurons in their own layer; E-neurons also project to the other layer. B. Layout of hypercolumns (squares) in L4 and L6. Each HC is divided into four subdomains (triangles) preferring a different orientation. Domains preferring the same orientation in adjacent HC are next to one another as shown. C. Layout of neuron groups. 7 × 7 groups of neurons in each hypercolumn, centered at the blue crosses.

Connectivities among the three components in the model are depicted schematically in Fig 2A. Drifting gratings are presented to the eye, and retinal responses are transmitted to the thalamus. We do not model retinal responses but start from the LGN, the response of which is believed to closely reflect that of the retina. The LGN component of our model projects directly to both L4 and L6. These two cortical layers communicate with one another but are not assumed to project back to LGN.

Each of the two cortical layers is divided into hypercolumns (HC). Following the design in [6], each HC is represented by a square, and it is further subdivided into intended orientation domains; see Fig 2B. We model a patch consisting of an array of 17 × 17 HC, each one of which corresponding to an area of 0.25° × 0.25° in the retina. Each HC is divided into four orientation domains consisting of neurons favoring edges at 0°,45°,90° and 135°; 0° and 90° are sometimes referred to as vertical and horizontal respectively. Within each HC, we divide the E-population into 49 groups arranged in a 7 × 7 lattice; see Fig 2C. Since there are roughly 3000 E-neurons in a HC, each group consists of ∼60 E-neurons. I-neurons are divided into the same number of groups and represented similarly; the number of I-neurons in each group is ∼20. For simplicity, we used the same 7 × 7 lattice for LGN, even though the number of LGN cells are in reality much smaller.

Hypercolumns in LGN and the two cortical layers are identified through their projections to the retina. Groups of neurons with the same receptive fields in the three components of the model are assumed to be vertically aligned.

In the study presented here, LGN cells are assumed to function as filters the properties of which are known and are summarized in Sect. 2.2.2. The entire model is assumed to be *linear* to begin with; nonlinearities are considered only later on. In the linear model, each group of neurons in the two cortical layers acts as an integrator, summing the excitatory and inhibitory currents it receives to produce an output: Let Input(*i* → *j*) denote the current transfer from Group *i* to Group *j*; it is positive or negative depending on whether Group *i* is excitatory or inhibitory. Then for Group *n* in either L4 or L6, we have

$$\begin{array}{c} \text{Output}\left(n\right)\;=\;\sum_{i}\text{Input}\left(i\to n\right) \end{array}$$ (1)

where Output(*n*) is the output of Group *n* (only solutions with nonnegative outputs are acceptable). Here the summation is taken over all groups in LGN, L4 and L6 that project directly to Group *n*. The term Input(*i* → *j*) is given by

$$\begin{array}{c} \text{Input}(i\to j)\;=\;\text{Output}(i)\;\times \;\text{Prob}(i\to j)\;\times \text{Wt}(i\to j)\;, \end{array}$$ (2)

where Prob(*i* → *j*) is the connection probability from Group *i* to Group *j* and Wt(*i* → *j*) is the synaptic coupling weight, its sign depending on whether Group *i* is excitatory or inhibitory. These quantities are discussed in Sects. 2.2.3 and 2.2.4. Outputs of LGN come from explicit integration of functions representing external stimuli, i.e., drifting gratings of different sizes. Combining Eq (1) for all groups *n* in L4 and L6, one obtains the equation that gives the outputs for all groups in L4 and L6 defining the linear model. See Methods for details.

#### 2.2.2 LGN outputs

In real neurobiology, LGN cells respond to changes in luminance and cortical neurons acquire their orientation selectivity through the spatial alignment of their ON and OFF LGN afferents. In this paper, we will bypass the detailed relationship between LGN and cortex, replacing it by simplified rules. This is adequate because all that concerns us is the mean input LGN cells at the various locations provide for neurons in L4 and L6 in response to gratings of different sizes.

Below and in the rest of the paper, all length measurements are in degrees, and the unit is often omitted. A drifting grating of radius *R* at full contrast is represented (abstractly) as a function

$$\begin{array}{c} g_{R}\left(z\right)=\{\begin{array}{c} 1\;\text{for}\;|z|\leq R\;,\\ 0\;\text{for}\;|z|>R\;. \end{array} \end{array}$$

Here *z* denotes a location in the retina (LGN or cortex). The center of the grating is assumed to be located at 0, and |*z*| is the distance from 0, measured in degrees.

**Fig 3:**
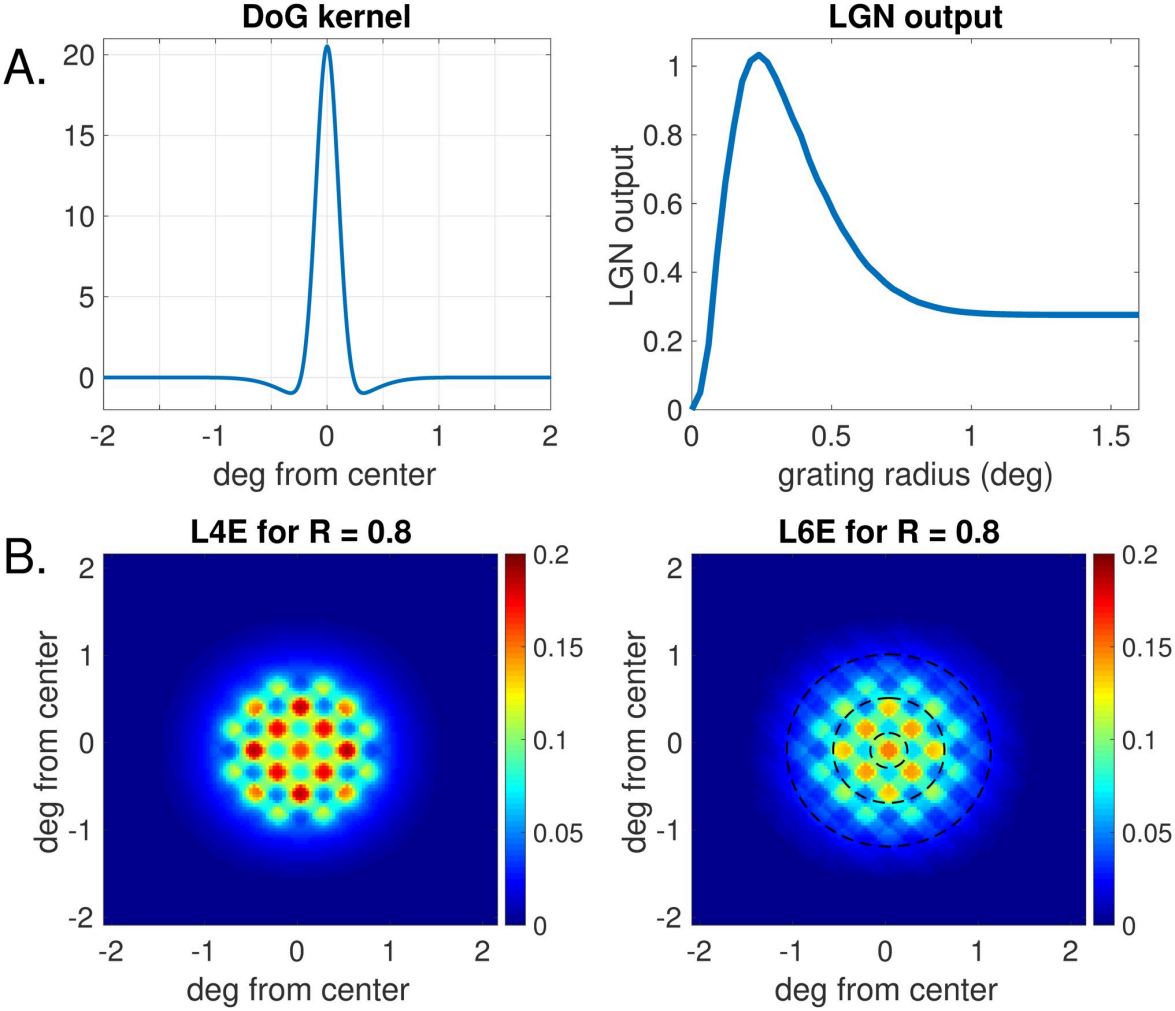
A. LGN responses to visual stimuli. Left: The difference of Gaussians (DoG) kernel. Right: LGN outputs at the center as a function of the grating radius. B: Activity maps showing outputs of model neurons in response to a grating of size *R* = 0.8. The grating has vertical stripes, and is at high contrast. Brightly colored diamonds correspond to vertical-preferring domains. Left: Outputs of E-neuron groups in L4. Right: E-neuron groups in L6. See Section 2.4.1 for the meaning of the dashed circles in the right panel.

LGN response to drifting gratings are depicted in Fig 3A. To model center-surround effect, the response of each LGN group is obtained by integrating the stimulus function *g*_*R*_(*z*) against a kernel given by the difference of two Gaussians (DoG), the positive one with SD = 0.1 and the negative one with SD = 0.31, as shown in Fig 3A, left. Here LGN output in background is set to 0. Given a drifting grating of radius *R*, strictly positive outputs are produced by LGN groups located in a disk of radius slightly larger than *R*. The panel on the right records the response at the center of the grating as a function of *R*.

In the real visual cortex, neurons in optimally driven orientation domains receive LGN inputs that differ in firing pattern than those received by neurons in orthogonally driven domains; the amounts of current transferred are identical; see e.g. [6]. Since firing patterns are not modeled here, we use the following rule to simulate the corresponding effect: Suppose LGN input to a group of E-neurons in L4 or L6 at location *z* computed using Eq (2) is *w*, and this group prefers orientation *θ*, *θ* = 0°, 45°, 90° or 135°. In response to a grating with orientation *θ*_0_, we assume

$$\text{LGN-input}\;\text{to}\;\text{the}\;\text{group}=\{\begin{array}{ll} w & \text{if}\;\theta =\theta_{0},\\ 0.4w & \text{if}|\theta -\theta_{0}|=45^{{}^{\circ}},\\ 0.1w & \text{if}|\theta -\theta_{0}|=90^{{}^{\circ}}. \end{array}$$

This rule applies to groups of E-cells. I-cells are not assumed to receive orientation information directly from LGN, so LGN input at a specific location is independent of orientation preference.

#### 2.2.3 Connectivity

Connectivities between distinct groups of neurons are encoded in the quantities Prob(*i* → *j*), the probability of group *i* projecting to group *j*. These probabilities depend on (a) source and target neuron types (i.e. E to E, E to I, I to E or I to I) and (b) the distance between the two groups; they do not depend on where in cortex these groups reside. We will treat separately connection probabilities within local circuits and for long-range projections. Within local circuits, there is a fair amount of guidance from anatomical papers and from previous modeling work [6]. In this study, we will assume these connection probabilities are known and follow previously established values; a summary is provided below and details are given in **Methods**. For long-range projections, experimental guidance is inadequate, and we will identify below what is missing.

*Local circuits:* For *i* fixed, Prob(*i* → *j*) as *j* varies is described by a (normalized) truncated Gaussian the variance of which may depend on layer or neuron type. The same rule applies to inter-laminar projections assuming we identify the locations of the two layers. Spatial extents of the projections for different types of neurons, and the radii at which the Gaussians are cut off, are given in Table 1 below. More information is provided in Methods.

**Table 1:** List of key parameters for connections within local circuits and between L4 and L6. “*x*/*y*” in the last column means that the probability of connection to group *i* is given by a normalized truncated Gaussian centered at *i*, with SD = *x* (in degrees), and cutoff at *y* SD. Parameter values in this table are roughly equivalent to those in [6], which are constrained by anatomy and data. Model parameters not listed here are to be determined.

Source	Target	Coupling weight	Spatial extent (SD)/cut-off
LGN	4E	0.3	0.2 / 1 SD
	4I	0.25	0.2 / 1 SD
	6E	0.12	0.2 / 1 SD
	6I	0.12	0.2 / 1 SD
4E	4E	1.44	0.077 / 2 SD
	4I	0.95	0.077 / 2 SD
	6E	0.42	0.06 / 1 SD
	6I	0.75	0.06 / 1 SD
4I	4E	0.9	0.055 / 1.5 SD
	4I	0.7	0.055 / 1.5 SD
6E	4E	Control	0.07 / 2 SD
	4I	0.81	0.07 / 2 SD
	6E	0.94	0.075 / 2 SD
	6I	0.96	0.075 / 2 SD
6I	6E	0.7	0.05 / 2 SD
	6I	0.7	0.05 / 2 SD

*Long-range projections:* It is known that L6 E-neurons can make contact with neurons up to several hypercolumns away, and it is believed that they predominantly connect with neurons having like orientation preference, a property we have assumed. We are not aware of experimental data on the probabilities of connection as functions of distance, or how the probabilities of connection from E to E compare to those from E to I. These quantities affect surround suppression, and one of our challenges is to reverse-engineer them from observed surround properties.

#### 2.2.4 Coupling weights

In this paper, because we consider only groups of neurons (and not individual neurons), certain quantities have to be interpreted a little differently. In lieu of firing rates, we work with *current inputs* and *outputs* for each group, and *current transfers* between groups. The output of a group, which is defined to be the total current produced by that group, incorporates information on not only the group’s firing rate but also the number of neurons in the group.

As mentioned in Sect. 2.2.1, Eq (2), current transfer from Group *i* to Group *j* is defined to be

Input(i→j)=Output(i)×Prob(i→j)×Wt(i→j).

Unlike connection probability, which depends on the distance between the two groups, synaptic coupling weight which we now discuss depends only on neuron types (E or I, LGN, L4 or L6).

Within each layer, we were able to locate reasonable values for many of the coupling weights needed following [6], which contains data-based information deduced from either experiments or simulations. Coupling weights between the various groups are listed in Table 1 below. More information is given in Methods.

We identify below two quantities for which experimental guidance is inadequate:

*Coupling weights from L6 to L4:* Only specific types of L6 neurons project to L4 [8], and we are not aware of empirical information on their synaptic weights, or the relative weights to E and I-neurons in L4. These quantities are to be determined.

This completes a basic description of what we will call “*the linear model*”.

Before going further, we would like to acquaint the reader with our cortical activity maps, two examples of which are presented in Fig 3B. These panels depict the Excitatory outputs of the model in response to a drifting grating. The left and right panels represent the regions of L4 and L6 modeled. A vertical grating the center of which coincides with the point labeled (0, 0) in each panel is presented. Colors represent the sizes of the outputs above background; these numerical values by themselves have little significance since all are scalable: only relative values across the cortical surface have meaning. The diamond-shaped regions with larger outputs (yellow to red) are vertical-preferring and are therefore optimally driven. The grating has full contrast and radius 0.8 degree. The right panel shows that for L6, the center diamond has the largest output at *R* = 0.8, but in the left panel, the center diamond has slightly lower output than surrounding vertical-preferring diamonds, suggesting that for L4 center response likely peaked at a smaller radius. Note also that the region with above-background output is larger for L6 than for L4, due to the presence of long-range connections. We are aware that at this point we have not provided full information about the model; Fig 3B is intended mostly to give the reader a preliminary view of the spiking activity across the two cortical layers in response to a drifting grating.

**Summary of Section 2.2:** We have presented in this subsection a partially constrained model of the LGN-L4-L6 circuit of the visual cortex. A priori constraints on (i) LGN responses and (ii) parameters pertaining to local circuits in L4 and L6 are gleaned from experimental data and previous modeling work; their approximate values are assumed to be known. Crucial to the phenomenon of surround suppression but for which experimental guidance is lacking are

(a) long-range connection probabilities within L6, and
(b) coupling weights from L6 to L4.

These quantities are to be determined.

### 2.3 Experimental data and computational problem

In Sect. 2.3.1, we set targets for our model and discuss supporting experimental data. In Sect. 2.3.2, we lay out the problem in terms the model presented in Section 2.1, and outline the computational procedure for going forward.

#### 2.3.1 Data on size-tuning curves and suppression indices

As discussed in Sect. 2.1.1, one of the most basic forms of surround suppression is the following: One presents to the eye a series of drifting gratings of increasing size centered at the receptive field of an excitatory neuron. Assuming that the gratings are all aligned with the neuron’s orientation preference, there will be substantial spiking activity above background levels in response to the gratings. A phenomenon that has been observed and well documented is that the firing rates of the neuron at the center vary with grating size. Its firing rates (measured in number of spikes per sec) plotted as a function of grating radius *R* is what we will refer to as the *size tuning curve* for this neuron.

Size-tuning curves are the experimental data we will try to emulate. Specifically, we will use data for monkey, whose visual cortices have much similarity to human’s. It is generally accepted that the firing rate of a neuron at the center increases with *R* initially, peaking at some point, then decaying as *R* continues to increase, eventually leveling off; see the review article [1]. We will focus on responses to drifting gratings where the gratings are of a single orientation and the contrast is strong. Other kinds of gratings have been studied experimentally; they are used to demonstrate more refined properties of surround suppression, and we will not consider them here.

**Fig 4:**
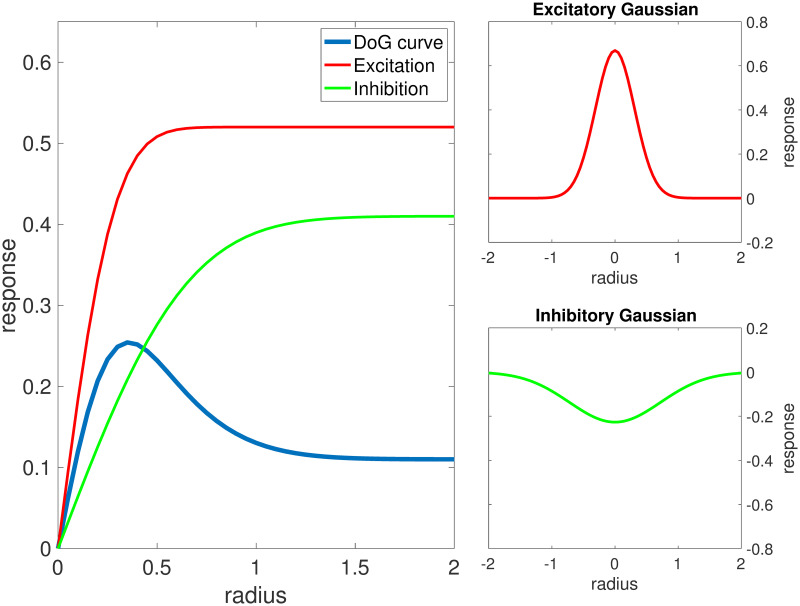
A DoG model. Red curve in the left panel is area from 0 to *R* under the positive Gaussian on the right (also red); green curve in the left panel is area above the negative Gaussian on the right; blue curve is the difference between red and green. For the DoG curve shown, SI = 0.5672 and SI_DoG_ = 0.7885.

Size-tuning curves have been shown to have very good fit with functions defined by differences of Gaussians (DoG) [11]; see Fig 4. For monkey V1, fairly detailed data on size tuning have been collected, including studies that treat laminar differences [11]. We will try to reproduce size-tuning curves for L4 and L6. Instead of measuring firing rates of individual neurons, however, we will measure outputs of groups of neurons located at the center. The groups in our model (see Sect. 2.2.1) are small enough that their outputs accurately reflect averages of spike rates for individual neurons for gratings of radius *R* ≥ 0.1.

As a quantitative measure of the degree of suppression, we will use a quantity called *Suppression Index* (SI), which can be thought of as approximately

$$\begin{array}{c} 1\;-\;\frac{\text{asymptotic}\;\text{firing}\;\text{rate}\;\text{for}\;\text{large}\;R}{\text{peak}\;\text{firing}\;\text{rate}}. \end{array}$$ (3)

Notice that SI ∈ [0, 1]; the closer it is to 1, the stronger the suppression.

The following are targets for E-neurons we will aim for, with justification to follow:

(i) the size-tuning curve for L4 should peak at *R* ∼ 0.4, and the one for L6 should peak at 0.7 − 0.8 (cf that for LGN, which in the model peaks at *R* ∼ 0.25); for L4, a substantial amount of the decline in firing rate at the center should have occurred by the time *R* reaches 1.0 − 1.5;
(ii) the profiles should be roughly approximable by DoG; and
(iii) our target values of SI (as defined in Eq (3)) are 0.4 − 0.5 for L4, and 0.2 − 0.4 for L6.

Two notions of SI are in use in the neuroscience literature. One is Eq (3), sometimes with a specified value of *R* in the place of “asymptotic firing rate”; see e.g. [12]. The other—let us call it SI_DoG_ for purposes of the present discussion—is defined in terms of the two approximating Gaussians; see [11]. These two values can be quite different, with SI < SI_DoG_; see the example in Fig 4. We have elected to go with SI, in part because it is easier to interpret, but mostly because the determination of best-fitting Gaussians in experiments used a much larger range of grating radius *R* than our model is able to capture, and behavior at these larger *R* can affect nontrivially the result of best DoG approximations. (With ∼300 hypercolumns, our model is able capture responses to gratings of radius up to *R* = 1.6°. Model complexity and simulation time grows rapidly with system size).

We explain how we arrived at our target list above. The peak values in Item (i) were gleaned from the following sources: LGN is from [7], L4 and L6 were deduced from [11]. The idea of (ii) is from [11]. As for target SIs, it is reported in [12] (see Figure 9 of [12]) that the mean SI as defined in Eq (3) with *R* = 4° in the place of “asymptotic value” is 0.4 − 0.5 for layer 4C*α* and 0.3 − 0.4 for L5/6, counting only neurons with surround suppression. As to how these values compare with the SI_DoG_ values reported in [11], not enough information is provided in [11] to allow the reader to convert one measure to the other for individual neurons. Using averaged values to do the conversion, the numbers translate to SI = 0.4 − 0.5 for L4 and 0.1 − 0.3 for L6 depending on whether one counts neurons that show no suppression. These should be taken as very rough estimates only, but they are not inconsistent with the values in [12]: It has been reported that magno-recipient and parvo-recipient neurons in L6 reside in quite distinct networks, and that the magno-recipient neurons have stronger surround suppression than parvo-recipient ones [13]. Results for these two groups are lumped together in [11], but since the magno-recipient network is the one that interacts with L4 hence the one we are modeling, the SIs of its neurons are likely closer to 0.3. Taking these facts into consideration, we have set our target SI for L6 at 0.2 − 0.4.

We note in advance that because our model can capture responses up to *R* = 1.6° only, and size-tuning curves may not have completed their descent at this radius, the SI produced by our model may be lower than targeted values.

The discussion above was all for E-neurons, which have been the primary target of experimental studies because their responses are easier to measure and they are the ones that project to other layers and brain regions. It was previously thought that I-neurons did not have surround suppression, and that the mechanism for surround suppression was that I-firing rate would continue to rise as *R* increases thus suppressing the E-neurons in the local circuit. These ideas have been refuted by more recent findings, which showed that I-neurons also have some amount of surround suppression, though less than E-cells [14]. We would like our model to emulate this behavior as well.

#### 2.3.2 Computation problem and procedure

As stated in the Introduction, the aims of this paper are twofold: One is to build a model that will emulate the behaviors of size-tuning curves as discussed in Sect. 2.3.1. The other is to use the model to shed light on the *mechanisms* behind the phenomena observed. For example, what makes L6 peak so late, after LGN firing rate at the center has undergone most of its decline? Why does L4 peak later than LGN? If L4 is influenced by L6, how come its center response starts to fall when firing rate at corresponding location in L6 is still climbing? These are some of the questions we hope to address.

As summarized at the end of Section 2.2, the situation is that we have constructed a model for which many of the parameters (namely those related to LGN and to local circuits in L4 and L6) have been constrained though there is some small amount of flexibility, but there is still a collection of parameters, namely those related to long-range connection probabilities in L6 and to coupling weights from L6 to L4, for which experiments have provided little guidance. Our next task, the results of which are reported in Section 2.3, is to locate these not-yet-determined parameter values, with the aim of producing a model that meets the conditions laid out in Section 2.3.1.

As explained in the Introduction, our approach is to first work with a *linear model*, for which simulation times are much shorter, and to go to a nonlinear model only after the linear model is found to be inadequate. Below we outline the procedure for selecting viable candidates for the yet unknown parameters.

We fix an orientation for the grating and a location in L4 (hence also L6) to be designated as “center”. All gratings below will be so oriented, and all are centered at this location. For each fixed set of parameters. the size-tuning curves for L4 and L6 are computed as follows: For a grating of radius *R*, we first compute the LGN response (Sect. 2.2.2), and plug that into the linear system defined by Eq (1) and Eq (2) in Sect. 2.2.1. Here the unknowns are the outputs of each of the E and I groups in L4 and L6 (see Sect. 2.2.1); more detail is given in S1 Text. This is repeated for a number of values of *R* between 0.1 and 1.6 degrees. The size-tuning curves are graphs of *f*_0_(*R*) where *f*_0_(*R*) is the output at the center in response to the grating of radius *R*.

Described in the last paragraph is the computation of size-tuning curves corresponding to *a single set of parameters*. As discussed above, there were many parameters to be constrained, such as long-range connection probabilities as a function of distance between the two groups of neurons in L6. The number of unknown parameters can be thought of as being on the order of 15 − 20. As is well known to be the case, it is impossible to systematically explore a parameter space of such dimensions. The way we arrived at the solution presented in the next subsection consists of (i) initial guesses based on *a priori* knowledge of model behavior, together with (ii) a series of steps in the spirit of a gradient descent guided by (iii) model analysis at each step.

We elaborate on (ii) and (iii). What was done in (ii) is not a gradient descent in the technical mathematical sense because the complexity of the situation makes it very challenging to formally design an objective function or to assign weights to errors or deviations from the objective function. Instead we determined the next step from the following model analysis. After each trial, we examined the resulting size-tuning curves to identify what is unsatisfactory about them. To understand what caused the undesirable features, we performed what we call a “*weight analysis*” two examples of which are presented in Sect. 2.4.3. Once the problems were identified, we proceeded to fix them by varying certain of the parameters. There are generally multiple ways to shift parameters to achieve any one single effect. Some parameter changes may introduce other undesirable effects and some are more sensitive than others. A second tool that we used is a *sensitivity analysis*, which amounts to computing the partial derivatives of outputs with respect to changes in individual parameters. The hope was that via a sequence of small changes, one would gradually steer the parameters towards desired goals.

The model analysis described in the last paragraph was semi-systematic; there was a learning curve but our ability to steer the model improved over time. An advantage of the method we used was that we learned, through the analysis performed, model dynamics and the underlying mechanisms responsible for the surround effects observed. Unraveling the mechanisms of surround suppression is one of stated goals of the present study. While the use of an artificial neural network (or other machine learning approach) seemed attractive, we found that it is very difficult to generate a meaningful training set. A randomly chosen set of unknown parameters has very low probability to produce a useful tuning curve. And generating a tuning curve itself is computationally expensive. Designing a suitable objective function is challenging as well. To make the machine learning-based parameter tuning work, a lot of human involvement in generating training sets and choosing loss functions would be necessary. This requires similar or more understanding to the model dynamics and the underlying mechanism of size tuning. We plan to do it in a separate project.

### 2.4 Results for the linear model: Parameter selection, model analysis and mechanistic explanations

In Sects. 2.4.1 and 2.4.2, we report on the best parameters we found following the procedure outlined in Sect. 2.3.2. These are not necessarily the only viable solutions to the problem, but we will demonstrate that locally, meaning among nearby parameters, they are close to being optimal. Mechanistic explanations are an equally important part of this section. In Sect. 2.4.3, we present two examples of weight analysis. As we will show, analyses of this kind offer a great deal of information on what goes on within the model. They were used to assist in much of our parameter search.

#### 2.4.1 Long-range connectivity in L6

Long-range connection probabilities in L6 hold the key to surround properties, for they determine how information in the surround is communicated to the center. We report here on what we found as we sought to locate these probabilities. We assumed from the outset the following:

(i) E-neurons, and only E-neurons, in L6 make connections beyond the local circuit, and they connect only to groups with like orientation preferences;
(ii) they contact both E and I-neurons;
(iii) the probability of connection between two groups is a function of the distance *d* separating the groups, and it decreases as *d* increases;
(iv) coupling weights between E to E, and E to I, will be the same for long as for short-range connections.

The first two items are consistent with known neuroanatomy. The last two seem reasonable and natural.

The two functions of interest, therefore, are *p*_*E*_(*d*) and *p*_*I*_(*d*), where *d* denotes the distance between the two groups of neurons in question, *p*_*E*_(*d*) is the connection probability when both presynaptic and postsynaptic groups consist of E-neurons, and *p*_*I*_(*d*) is when the postsynaptic group consist of I-neurons. In the discussion to follow, it will be assumed implicitly that all pre- and postsynaptic pairs considered have the same orientation preference, and we consider only *d* > 0.15, the size of the local circuit.

**Fig 5:**
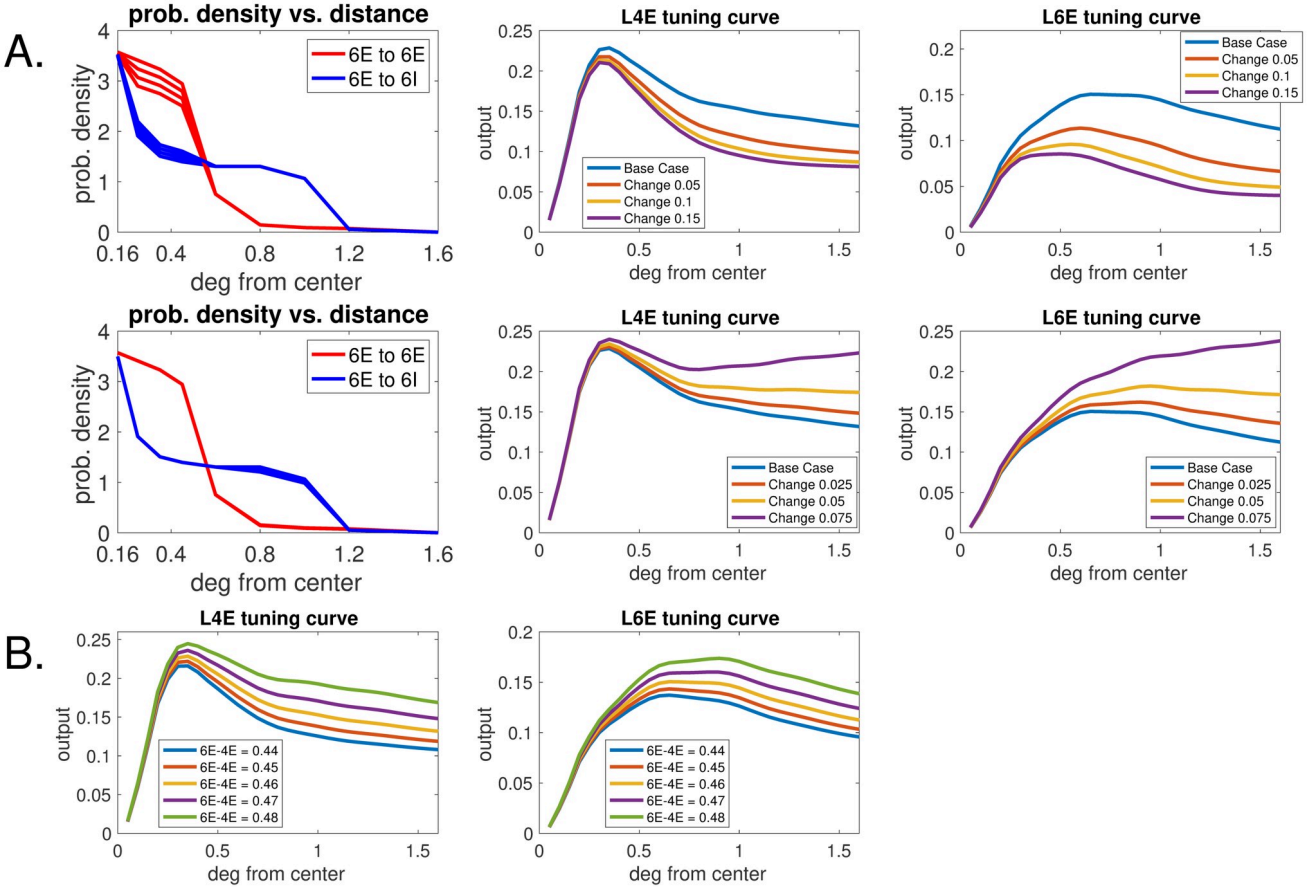
Size-tuning curves for selected parameters. **A.** Long-range connection probabilities in L6: the functions *p*_*E*_(*d*) and *p*_*I*_(*d*). **Top row.** Varying *p*_*E*_(*d*) and *p*_*I*_(*d*) for *d* ≤ 0.6. Left: *p*_*E*_(*d*) (red, uppermost) and *p*_*I*_(*d*) (blue, bottom) together with three variations in which *p*_*E*_(*d*) is decreased by 5%, 10%, and 15% and *p*_*I*_(*d*) is increased by the same percentages for *d* ≤ 0.6. Middle: L4 size-tuning curves for E-cells for each of the 4 sets of *p*_*E*_(*d*) and *p*_*I*_(*d*). Right: The same for L6. **Second row.** Varying *p*_*E*_(*d*) and *p*_*I*_(*d*) for *d* ≥ 0.6. Left: Similar to Top row; here *p*_*I*_(*d*) (blue, uppermost) and *p*_*E*_(*d*) (red, bottom) are decreased/increased by 2.5%, 5%, and 7.5% for *d* ≥ 0.6. Middle and Right: L4 and L6 size-tuning curves for the 4 sets of *p*_*E*_(*d*) and *p*_*I*_(*d*). **B.** Size-tuning curves for 5 6E-to-4E coupling weights (with 6E-to-4I coupling weights fixed). Parameters for the middle curve in B are identical to baseline parameters in A.

We performed simulations that amounted to partial parameter sweeps, and found that connection probabilities shown in Fig 5 (left) gave the best results. The two functions *p*_*E*_(*d*) and *p*_*I*_(*d*) we found have a rather striking relationship: For smaller values of *d*, the interaction is “net-excitatory”, meaning *p*_*E*_(*d*) is significantly larger than *p*_*I*_(*d*), and for larger *d*, the interaction is “net-inhibitory”, meaning *p*_*I*_(*d*) is significantly larger than *p*_*E*_(*d*). The cross-over occurs at around *d* = 0.6.

We made no prior assumption on the nature of the functions *p*_*E*_(*d*) and *p*_*I*_(*d*); they came out of the search. As is well known to be the case, it is impossible to search the entire parameter space. Without claiming that these are the only viable parameters, we present simulation results and supporting analysis to show the undesirable effects of deviating from the findings above.

**The net-excitatory regime** *d* < 0.6: In the top row of Fig 5, the left panel shows several versions of the graphs of *p*_*E*_(*d*) (red) and *p*_*I*_(*d*) (blue). Viewing as baseline the two graphs that are farthest apart, i.e., *p*_*E*_(*d*) is the top curve and *p*_*I*_(*d*) the bottom, we studied the outcomes of bringing them successively closer, decreasing *p*_*E*_(*d*) by 5%, 10% and 15% and increasing *p*_*I*_(*d*) by the same percentages. For each of the 4 sets of parameters we show in the middle and right panels the size-tuning curves for L4 and L6, i.e. the outputs of E-cells at the center as functions of *R*.

Let us focus on the size-tuning curves for L6, which are more directly impacted by these connection probabilities. Two things are apparent: One is that outputs decreased with net excitation between adjacent domains: no surprise there. The other is that the peaks of the size-tuning curves moved steadily to the left when *p*_*E*_(*d*) and *p*_*I*_(*d*) were brought closer. The same phenomenon, less pronounced, can be seen for L4.

*Analysis:* Recall from Sect. 2.3.1 that the size-tuning curve for LGN peaks at *R* = 0.25 whereas the one for L6 is supposed to peak at 0.7 − 0.8. The fact that cortical size-tuning curves peak later can only be due to cortico-cortical interaction. Since local circuits are <0.15 in radius, what causes firing rates at the center of L6 to continue to rise beyond *R* ∼ 0.35 up to 0.7 must involve net-excitatory interaction beyond the local circuit. Lowering the net-excitation between adjacent domains therefore is expected to cause L6 to peak at a smaller *R*, and this is confirmed by the top right panel of Fig 5.

We conclude that the top two curves on the right panel of Fig 5 produce more suitable size-tuning curves for L6 than the bottom two.

**The net-inhibitory regime** *d* > 0.6: This is depicted in the second row of Fig 5. We found that *p*_*I*_(*d*) had to be much larger than *p*_*E*_(*d*), or the outputs at the center would not decrease as *R* increased. The middle and right panels show the outcome of attempting to bring the two curves a little closer. We varied the baseline values by smaller amounts than for *d* < 0.6 because of the larger area being summed over. Decreasing *p*_*I*_(*d*) by a few percent caused the suppression indices of both L4 and L6 to lower substantially. The shapes of the size-tuning curves became unacceptable with a 7.5% decrease.

*Analysis.* The discussion below pertains to L6, which is more directly impacted by these connection probabilities. We offer an explanation for why in order for firing rate at the center to decrease as we increase grating radius *R*, the interaction between groups at distances *d* > 0.6 apart has to be net-inhibitory.

Calling the reader’s attention to Fig 3B, remember that those lit-up diamond shape regions represent orientation domains preferring a fixed (vertical) orientation. Below, we consider only domain preferring the vertical orientation. For ease of reference let us recall those dashed circles in the right panel of Fig 3B and introduce the following language: We call the orientation domains surrounding the one containing the center as the “first ring”, and the domains just outside of the first ring as the “second ring”. With this terminology, domains in the first ring are located at distances *d* < 0.6 from the center, while domains in the second ring are at distances roughly 0.6 < *d* < 1.1 from the center. Below we compare the following three gratings: Grating 0, which stimulates only the center (the inner dashed circle in Fig 3B Right), Grating 1, which stimulates only the center and the first ring (the intermediate dashed circle in Fig 3B Right), and Grating 2 (the outer dashed circle in Fig 3B Right), which stimulates the center and the first two rings.

First, L6 center response is stronger to Grating 1 than to Grating 0, in real data as with the parameters we chose above in relation to the *d* < 0.6 case. This means the recurrent excitation between adjacent domains more than compensates for the deficit in LGN input to the center as we go from Grating 0 to Grating 1. We compare next the response to Grating 1 vs Grating 2. Groups in the first ring fire more in response to Grating 2 than to Grating 1 for the same reason as above. This means that the excitatory input from the first ring to the center is stronger for Grating 2. While LGN input to the center is weaker for larger gratings, the change from Grating 1 to Grating 2 is insignificant as LGN’s size-tuning curve has largely leveled off at the radius of Grating 1; see Fig 2A. Thus even with no direct input from the second ring, center firing rate would be higher or roughly the same as we go from Grating 1 to Grating 2. But experiments tell us that this firing rate should fall. To achieve this, the input from the second ring is necessarily net-inhibitory. This reasoning is consistent with the values of *p*_*I*_(*d*)>*p*_*E*_(*d*) for *d* > 0.6 that we found numerically.

#### 2.4.2 Coupling weight from 6E to L4

We now select coupling weights from E-neurons in L6 to E and I groups in L4. Since the current transfer to 4E and 4I will have effects that cancel one another, we fix the coupling weight to 4I, and vary that to 4E. A partial parameter search produced the results shown in Fig 5B. The effects of varying these findings by up to 10% are also shown.

The effect of a larger 6E to 4E current transfer (with 6E to 4I fixed) is relatively straightforward to understand: It causes the size-tuning curves for both 4E and 6E to be higher. That is because locally the model is a feedback loop between L4 and L6, each supplying only excitation to the other. Other things being equal, an increase in 6E to 4E coupling increases the recurrent excitation within this local feedback loop, causing both L4 and L6 to become more excited. This is consistent with what is shown in Fig 5B.

#### 2.4.3 Weight analysis

By weight analysis, we refer to the decomposition of the total input to a group of neurons according to source. Such analyses are quite revealing: they explain not only the response of the group in question but give a sense of model dynamics by informing on the activity of groups that project to it. They were routinely performed in our parameter tuning process to assess the performance of the various parameters. Here we show a few examples of such analyses to illustrate the type of information that can be gleaned from them.

**Fig 6:**
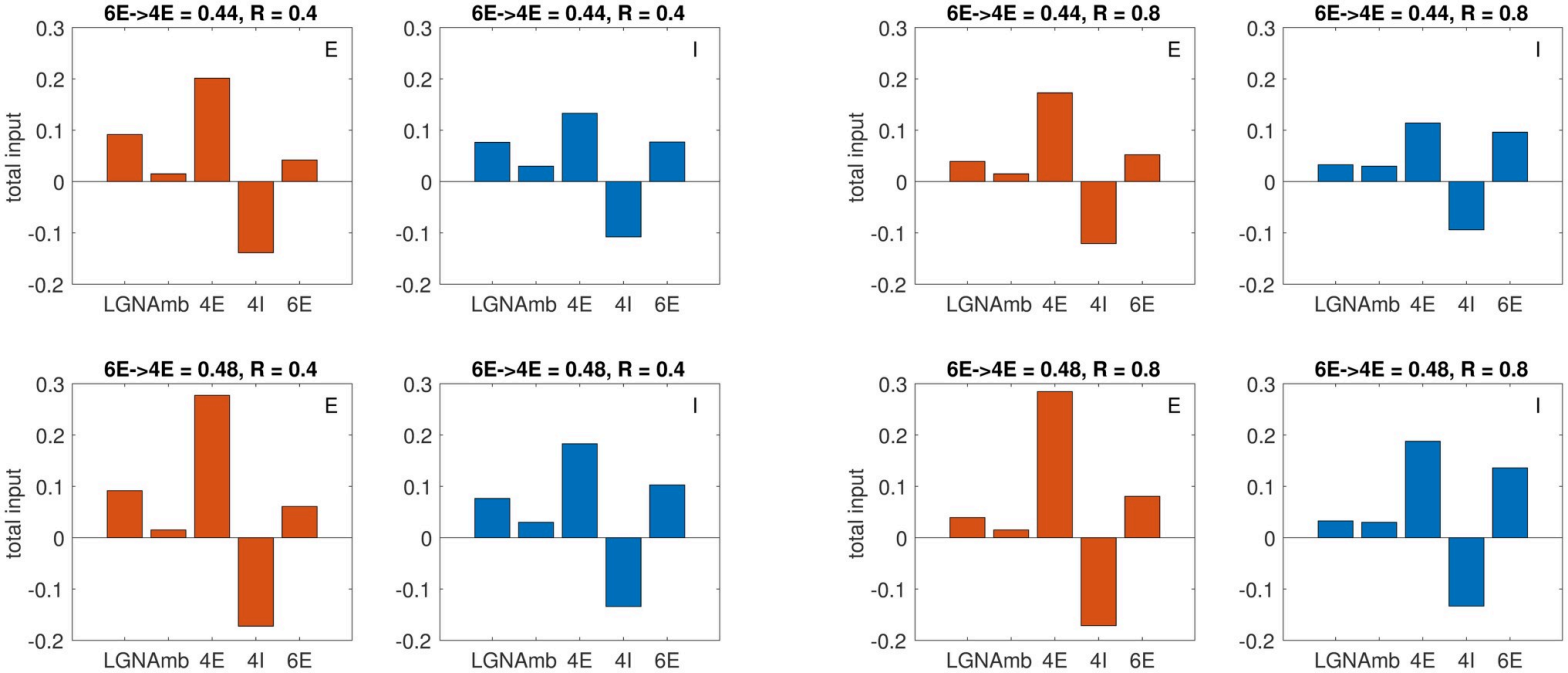
Weight analysis, i.e., breakdown of total input according to source to a group of E-neurons (red) and a group of I-neurons (blue) at the center in L4. Grating radius *R* = 0.4 for the group of 4 panels on the left, and *R* = 0.8 for group of 4 on the right. 6E-to-4E coupling weight = 0.44 (top row) and 0.48 (bottom row).

Fig 6 shows the weight analysis for two group of neurons, one E and one I, located at the center in L4. Shown are the current sources to these two groups under 4 sets of conditions: two different coupling weights of 6E-to-4E for two grating radii *R*. The following are examples of the type of information these plots offer:

(i) *Relative contribution of LGN, 4E and L6 to neurons in L4.* Recall that L4 has no long-range interactions, so in addition to recurrent excitation from within its local circuits, the only external input to L4 are from LGN and L6 (and a small amount of “ambient”, where we lump together all modulatory forces unmodeled). Both LGN and L6 are excitatory only. Looking at the group of E-neurons at the center (red bar graphs), we see that in all 4 examples, local 4E-to-4E recurrent excitation is by far the largest component. In particular, currents from lateral interaction are much larger than those from LGN. In this regard our model behavior is very much consistent with modern views of neuroscience (see e.g. [15, 16]), in stark contrast to earlier feedforward ideas.
(ii) *Recurrent excitation between L4 and L6.* We argued in Sect. 2.4.2 that increasing the synaptic coupling weight from 6E to 4E (while keeping that from 6E to 4I constant) will lead to higher firing in the center groups in both L4 and L6. Supporting evidence for this assertion can be seen clearly in Fig 6: contributions from 4E, 4I and 6E in the bottom row (where the coupling weights from 6E to 4E is larger) are all larger than in the corresponding panels above, and the increase in total excitatory input (from 4E and 6E) exceeds the increase in inhibitory input. This is true for both E and I-groups and at both *R* = 0.4 and 0.8. We deduce that the firing rates of 4E, 4I and 6E are all higher as a result of recurrent excitation in the L4-L6 loop at the center when coupling weight from 6E to 4E is increased.
(iii) *Swapping LGN for L6 and an explanation for the peak of L4 tuning curves.* Here we examine the shift in contribution to E-cells as we go from *R* = 0.4 to *R* = 0.8. In both rows, one sees clearly that the amount of LGN input goes down, while the amount of L6 input goes up. (LGN input drops from 0.092 to 0.039; 6E input increases from 0.042 to 0.052 in the first row, and from 0.061 to 0.080 in the second row, where 6E to 4E coupling is larger.) This suggests that a factor that contributes to the downturn of L4 size tuning may be the more rapid withdrawal of current from LGN, which peaks at a smaller *R* and declines fast, relative to the slower rise in L6 activity as *R* is increased. It is also consistent with the more rapid decline in the L4 curve in the top row relative to the bottom (Fig 5B).
(iv) *Net-inhibitory nature of L6-to-L4:* Observe that in all four sets of simulations shown, the current transfer from 6E to 4I is larger than that to 4E. Even in the bottom row, where 6E-to-4E is larger and the shape of the tuning curve is becoming questionable (Fig 5B), the 6E component to I is still significantly larger than that to E (at *R* = 0.4: 6E-to-4E has weight 0.061, vs 0.080 for 6E-to-4I; at *R* = 0.8, where L6-firing is higher: 6E-to-4E has weight 0.10, vs 0.14 for 6E-to-4I.) That the projection of L6 to L4 was net-inhibitory was not a choice we made; it was a result of our search, and it is consistent with data for mouse [17]. For monkey, to our knowledge no definitive empirical information is available at the present time.

**Summary of Section 2.4:** Our main findings can be summarized as follows:

(a) With regard to the probabilities of longer-range connections within L6, we found through our parameter search—and also through an independent analysis—that to produce results consistent with data, *interaction between adjacent domains with the same orientation preference has to be net-excitatory, while interaction between domains two or more hypercolumns apart has to be net-inhibitory.*
(b) A plausible explanation for why the L4 size-tuning curve peaks after that for LGN and before that for L6 is that as *R* increases, L4’s current supply from LGN degrades more rapidly than the increase in current supply from L6.
(c) Our parameter search suggested strongly that *L6-to-L4 projections are net-inhibitory. Increasing this net-inhibition* by decreasing the coupling weight from 6E to 4E (without changing that from 6E to 4I) *will lower firing rates in both L4 and L6*.

The findings in (a), (b) and (c) above are all **testable model predictions**.

### 2.5 Introduction of nonlinearity

As explained earlier, our approach was to first work with a linear model, and to go to a nonlinear model only after the linear model was found to be inadequate. This approach has two advantages: One is that simulation times for linear models are much shorter, which is important because parameter selection is an interactive process that involves some amount of trials and errors. The second advantage is that a linear model will reveal where nonlinearities might be needed, and offer baseline parameter values to start from.

This subsection is not intended as a systematic study of how nonlinearities can lead to improvements in the model, but rather to present an example to demonstrate the viability and effectiveness of this approach.

#### 2.5.1 Issue with linear model and proposed solution

While our linear model produced size-tuning curves that roughly matched those seen in data, they were less than perfect. For example, for gratings of radius between 0.5 − 1.0, it was hard to keep L6 center response elevated and at the same time to have L4 center firing rate fall as steeply as we would like. We demonstrate below how this can be improved with the addition of a suitably chosen nonlinearity.

To better illustrate the problem observe in the top row of Fig 5A that either the L6 size-tuning curve peaked at too low a value of *R* (the bottom 3 curves), or the parameters led to a L4 curve that descended roughly linearly for 0.6 < *R* < 1.6, inconsistent with a DoG description. To force the L4 curve to descend more rapidly, one needs to decrease 6E to 4E (as LGN levels off after a while), and we see the result of that in Fig 5B: the L4 curve did go down more steeply, but decreased recurrent excitation between L4 and L6 lowers L6 firing rate (Sect 2.4.3) leading to the L6 curve peaking at a lower *R*.

We pinpoint the underlying difficulty as follows: For *R* < 0.6°, we would like L6 firing rate to rise with *R*, and to do so by taking advantage of the recurrent excitation between L4 and L6. For that to work well, L6 should not be too strongly net-inhibitory. For larger *R*, the more net-inhibitory the projection from L6 to L4, the more effectively it suppresses L4. But in a linear model, synaptic coupling weights between two groups of neurons are fixed once and for all; they cannot be adjusted depending on stimulus. This is what motivated the proposal below:

**Proposed nonlinearity:** *We hypothesize that the synaptic coupling from 6E to 4I is larger when 6E firing rate exceeds a certain threshold.*

There is biological justification for this, namely that some types of I-neurons (e.g. I-neurons of SOM type) are known to have a higher threshold but once activated they spike at high rates [18]. Such neurons are in the minority in Layer 4C*α*, but are thought to be present. Since we do not model different types of I-neurons, the action of SOM neurons can be modeled as an increase in the synaptic coupling between 6E and 4I when L6 is excited above a certain threshold.

We conjectured that this nonlinearity would help remove the undesirable feature identified above because L6 firing rate at the center is high around *R* = 0.5 − 1.0, exactly where we would like the L4 size-tuning curve to experience a more precipitous decline. Simulation results with this added nonlinearity are presented in the next subsection.

#### 2.5.2 Results from nonlinear model

Recall that among the suitable regimes we found for the linear model, coupling from L6 to L4 are to varying degrees net-inhibitory (Sect. 2.4.3, Item (iv)). Here we fix the 6E-to-4E synaptic coupling weight to be 0.48, the least net-inhibitory regime shown in Fig 5B, and add a nonlinearity as proposed above, by increasing the coupling weight by an amount proportional to the excess of 6E output beyond a certain threshold. We also add a small amount of nonlinearity to 4E-to-4E in the same spirit, to simulate the effect of recurrent excitation. Details are given in S1 Text.

**Fig 7:**
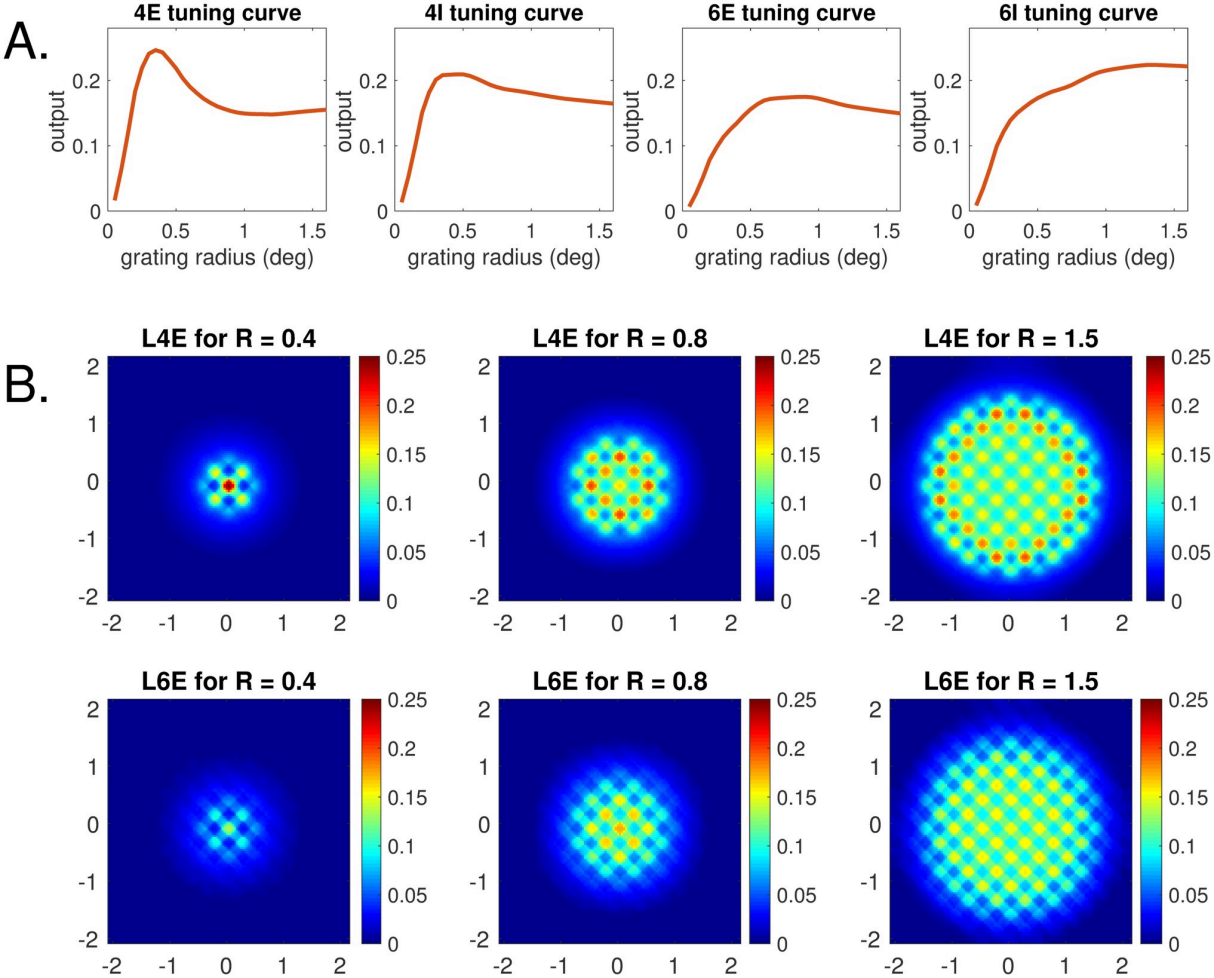
The nonlinear model. A. Size-tuning curves. The 4 panels are, from left to right, size-tuning curves for 4E, 4I, 6E and 6I respectively. B. Activity maps for the entire model. Top row: Excitatory groups in L4, bottom row: E-groups in L6. Responses are to gratings with R = 0.4, 0.8, 1.5.

Results of simulations as shown in Fig 7A are as anticipated: The size-tuning curve for 6E peaked between *R* = 0.5 − 1.0; the 4E tuning curve peaked earlier but also declined more steeply, having a shape more consistent with that of DoG (see e.g. Fig 4). Note also that 4I also has some amount of surround suppression though not as much as for 4E, consistent with the results of [14].

The three panels in Fig 7B show what are called *activity maps* in [16]. They tell us, at a glance, the outputs or levels of activity in all the E-groups in L4 and L6 in response to gratings of various sizes. For more detail on how to read these maps, we refer the reader to the last paragraph of Sect. 2.2. The color of the center pixels of these maps are data points from the size-tuning curves in Fig 7A. To our knowledge responses at locations other than the center have not been measured systematically in experiments. Activity maps like the ones shown in Fig 7B for real visual cortex are easy to obtain: one simply shifts the location of the grating relative to the receptive field of the neuron being recorded. These maps are **testable predictions** of our model, and they shed light on cortical dynamics; we propose the idea to experimentalists.

Finally, we remark on the practicality of the approach we took, namely to first work with a linear model and to add nonlinearities as needed to correct observed defects. The main parameter search here was for (i) the threshold of 6E output at which the nonlinearity started to kick in, and (ii) the amount of linear increase in 6E-to-4I synaptic coupling weight when 6E exceeded this threshold. We did the same for 4E-to-4E coupling but that was optional. All other parameters were from the linear model, and this relatively limited search made the problem much more tractable.

**Summary of Section 2.5:** Drawing inspiration from SOM-neurons, we explored a nonlinearity which elevates the coupling weight of 6E-to-4I when 6E firing rate is high, and that improved the overall quality of the L4 size tuning curve.

## 3 Discussion

The main scientific aim of this project was to obtain a clearer understanding of the mechanisms of surround suppression in input layers to the monkey V1 cortex. A secondary aim was to develop techniques of general interest in large-scale computational modeling of neuronal systems. We now discuss separately what we have been able to achieve with regard to these two aims, how our results compare to those in the literature, and what questions remain.

### 3.1 Overall assessment of model outputs

We have produced, using a single model with a fixed set of parameters, size-tuning curves that show good agreement with experimental data for E and I-cells in L4 and for E-cells in L6 (no known data for I-cells in L6 to compare to). Qualitative agreement with certain experimental data are excellent. They include (a) relative locations of the peaks of LGN, L4 and L6 tuning curves, and (b) Suppression Indices for 4E-cells being larger than those for 4I and 6E; (c) grating radii at which these peaks occurred were also realistic.

The model is not without limitations, however: our best parameters did not match perfectly the stated target values of SI. Part of this has to do with the fact that the range of *R* studied is not large enough (especially for L6, which peaks quite late); the simplistic modeling no doubt plays a role as well. Real neurons have much more complex behaviors than what one can hope to capture in simple mean-field models.

### 3.2 Mechanisms of surround suppression

Since there are no long-range connections within LGN or Layer 4C*α*, the only way neurons at the center can acquire information about properties of the stimulus outside of their classical receptive fields is via L6, the only layer in our 3-component circuit with long-range Excitatory connections. So we start by explaining why the size-tuning curves for L6 E-cells peak so late, long after the corresponding curve for L4 has declined and that for LGN has leveled off. To emulate data, which show that L6 size-tuning curves peak at around 0.7°, projections of E-groups within this radius to the center had to be net-excitatory (meaning they excite the E-cells at the center more than I-cells), and long-range projections from outside of this radius had to be net-inhibitory. We did not impose these parameter choices; we were led to them in our efforts to emulate data. Nor do we know if this switch from net-excitatory to net-inhibitory occurs in the real monkey cortex, or how it comes about if true (e.g. whether it has to do with the lengths of axons or the types of cells targeted).

Next we turn to Layer 4C*α*, the main input layer in the magno-pathway and also the main object of our investigation. It is natural that L4 peaks later than LGN, as the local circuit within L4 averages out the feedforward input L4 neurons receive, and the difference in peak radii between LGN and L4, which is around 0.15°, agrees well with the approximate radius of local circuits in L4. More curious is why the size-tuning curve for L4 drops off fairly steeply when the L6 curve is still climbing. Withdrawal of LGN input is likely responsible for the initial downturn, but LGN input levels off quickly. We found that an effective means to sustain the decline of the L4 curve is net-inhibition from L6, especially when that is increased with increased L6 activity (Section 2.5). These findings from our model analysis are related to neurobiology as follows: L6 to L4 projection is thought to be net-inhibitory for mouse [17]; for monkey it is not known and is a model prediction. Increased inhibition at elevated activity levels is consistent with the behavior of SOM neurons.

The results above, including the nature of the long-range coupling between L6 orientation domains as function of distance, and the net-inhibitory nature of the projection from L6 to L4, are part of our **model predictions.**

### 3.3 Relation to literature

Important as surround suppression is as a visual phenomenon, surprisingly few explanations for it have been put forward; see the recent, authoritative review article [1] on the topic. We discuss below three groups of of results that have received much attention.

One is [2], which offered a broad principle that suggests that surround suppression, like many other neural phenomena, comes from “normalization”; how normalization is performed in the brain is not discussed. [3] and [4] studied inhibition-stabilized and stabilized supralinear networks (SSN); surround suppression is mainly studied in [4]. These models are not constrained by data the way our model is, but we do have some qualitative features in common: in [4] the E-to-I footprint is larger than the E-E footprint, a feature similar to our finding in Sect. 2.4.1 that to emulate size-tuning data, shorter-range lateral interactions should be net-excitatory and longer-range interactions net-inhibitory. There are also many differences: [4] is about layer 2/3; surround effects inherited from previous layers were not taken into consideration, and the authors were primarily interested in demonstrating that SSN together with the connectivity above reproduced well experimental data such as contrast-dependence and oscillatory responses as function of stimulus size. Our study was focused on the input layers; it was a more biologically detailed study of only the most basic properties of surround suppression, and our goal was to explain underlying mechanisms.

Our results also resonated with those [5], which designed (without offering biological justification) a model in which suppression comes from a group of neurons external to the population. In our model, suppression in L4 came mostly from L6, especially in the nonlinear model, and was helped initially by the withdrawal of excitation from LGN. Suppression in L6 came from net-inhibitory couplings with domains two or more hypercolumns away. In both cases, suppression originated from sources external to the local circuit in question. These properties were emergent in our model, yet they mirror the picture proposed in [5].

Finally, we cannot stress enough the importance of *lengthscales* in surround studies, for they hold vital clues on mechanisms. We have tried to incorporate anatomical lengthscales in our model design, and have sought to respect lengthscales when reproducing experimental results in ways more than was previously done.

### 3.4 Towards systematic computational modeling of large neuronal systems

At ∼4000 neurons per hypercolumn per layer, the number of neurons is vast even in a fraction of the monkey V1 cortex. Computational models that simulate the dynamics of individual neurons become unwieldy quickly, and mean-field models cannot capture certain kinds of information. This paper explores a middle ground: we have chosen a version of local mean-field modeling in which neurons are grouped together for computational feasibility but the groups are small enough to distinguish orientation preferences and to permit recurrent excitation and inhibition to occur among the groups. Importantly, this moderate amount of coarse-graining permitted us to retain an accurate notion of lengthscale, an essential feature when comparing model outputs to physiological data.

Even for a local-mean-field model such as ours, the determination of parameters such as connection probabilities and synaptic coupling weights can be a daunting task. Yet biological realism, which includes suitable choices of parameters, is necessary if one’s aim is to shed light on cortical mechanisms. In this paper, we proposed—and demonstrated—a systematic way to build a biologically constrained model. The idea is to start from a linear system, and to “add” nonlinearities as needed. Such an approach has a number of advantages, two of which we have discussed earlier: One is that computationally it is much faster to solve a linear problem than to solve a nonlinear one by an iterative scheme (to give some indication of dimension, for the model in this paper the linear problem has a little over 50,000 variables). A second advantage is that we will learn where a linear model is inadequate, i.e., which phenomena are mediated by nonlinear interactions. We now add a third point, and that is: a simple linear model will reveal readily what experimental data are missing.

To illustrate this third point, consider the dynamics of long-range interactions, such as what we have in L6 of our model. Knowledge of long-range connection probabilities to E and I-groups, i.e., *p*_*E*_(*d*) and *p*_*I*_(*d*) as defined in Sect. 2.4.1, is crucial to our understanding of surround mechanisms. Assuming the model is linear, it is not hard to see that to solve for the functions *p*_*E*_(*d*) and *p*_*I*_(*d*), it would be helpful to know not just the firing rates of neurons at the center but those in vertical-preferring domains at other locations. Specifically, information on *f*_*E*_(*R*,*s*), the firing rates of E-groups at distance *s* from the center when a grating of radius *R* is presented, for *s* ≤ *R*+1 or so, would contribute to the constraining of *p*_*E*_(*d*) and *p*_*I*_(*d*). These firing rates are easy enough to measure, but to my knowledge such data have not been made available by any lab.

Now many neural phenomena including surround suppression are likely to have some nonlinear aspects. The next challenge would be to devise ways to assess nonlinearities and to properly incorporate them into the model. There are many other potential improvements. For example, different neuronal types and their synaptic dynamics can be “added” at a later stage. The methodology we propose is to start from something basic, go as far as one meaningfully can, and then upgrade systematically. It was in this spirit that we have carried out the project reported in this paper.

## 4 Methods

### 4.1 Linear model

#### 4.1.1 LGN input

Let Ω be the set of all lattice points given in Fig 2C. More precisely, we have

$$\begin{array}{c} \Omega =\{z=\left(x,y\right)\;|\;x=\frac{h}{2}+mh,y=\frac{h}{2}+nh,0\leq m,n\leq 118\}\;, \end{array}$$

where *h* = 1/28 (deg) is the distance between two adjacent neuron groups.

The LGN response is computed through the convolution of a difference of Gaussians (DoG) kernel and a grating function. For each *z* = (*x*,*y*) ∈ Ω, the grating function *g*_*R*_(*z*) takes value 1 for |*z*| ≤ *R* and 0 for |*z*| > *R*. The DoG kernel is given by

$$\begin{array}{c} f_{DoG}\left(z\right)=\frac{1}{Z}\{\frac{1+\Sigma }{2\pi R_{\text{in}}^{2}}e^{-{\left|z\right|}^{2}/2R_{\text{in}}^{2}}-\frac{\Sigma }{2\pi R_{\text{out}}^{2}}e^{-{\left|z\right|}^{2}/2R_{\text{out}}^{2}}\}\;, \end{array}$$ (4)

where Σ = 4.1 is the shape parameter, *R*_*in*_ = 0.1 and *R*_*out*_ = 0.31 represent standard deviations of the inner and outer Gaussian kernels respectively, and *Z* = 3.62283 is a normalizer that controls the maximal value of *f*_*DoG*_.

Let z* = (x*,y*)∈Ω be the coordinate of an LGN cell on the lattice shown in Fig 2C. The LGN response to a grating function *g*_*R*_(*z*), denoted by *LGN*(z*), is given by the following convolution equation

$$\begin{array}{c} \text{LGN}\left(z^{*}\right)=\max\{\int f_{DoG}\left(z-z^{*}\right)g_{R}\left(z\right)dz,0\}\;. \end{array}$$ (5)

In our computation, this integral is approximated by summing up *z* ∈ Ω.

#### 4.1.2 Connectivity and coupling weight

In local circuits, the connectivity between two groups of neurons (LGN, 4E, 4I, 6E, 6I) is given by a truncated 2D Gaussian kernel. The truncated Gaussian is then multiplied by the synaptic coupling weight that is independent of location. L6 E-neurons have long range connections, which will be specified later. Take 4E-to-4E connections as an example. The coupling between two groups of neurons with coordinates *z* and z* in Ω is given by

$$\begin{array}{c} q_{4E4E}\left(z,z^{*}\right)=\frac{h^{2}W_{4E4E}}{2\pi \sigma_{4E4E}}e^{-|z-z^{*}{|}^{2}/2\sigma_{4E4E}^{2}}1_{\left\{|z-z^{*}|\leq R_{4E4E}\right\}}\;. \end{array}$$

Here *W*_4*E*4*E*_ is the coupling weight, *σ*_4*E*4*E*_ is the standard deviation of the Gaussian kernel, and *R*_4*E*4*E*_ is the radius of the footprint of the 4E-to-4E connection. Other coupling weights are given analogously.

As shown in Fig 2A, 4E and 4I receive connections from LGN, 4E, 4I, and 6E; 6E and 6I receives connections from LGN, 4E, 6E, and 6I. See Table A-E of S1 Text for the full list of parameters. The L6-to-L4 coupling weight *W*_6*E*4*E*_ is a control parameter. We choose different values of *W*_6*E*4*E*_ from 0.44 to 0.48 in our simulations.

In addition to local connections, 6E neurons also have long-range connections to L6 neuron groups with the same orientation preferences. We assume local circuits have radius 0.15. Setting *p*_*E*_(0.15) = *p*_*I*_(0.15) = 1.0, the coupling weight between two L6 E-groups with the same orientation preferences and located at *z* and z* with |*z* − z* | > 0.15 is

$$\begin{array}{c} q_{6E6E}\left(z,z^{*}\right)=q_{0}p_{E}(|z-z^{*}\left|\right)=\frac{h^{2}W_{6E6E}}{2\pi \sigma_{6E6E}}e^{-R_{6E6E}^{2}/2\sigma_{6E6E}^{2}}p_{E}(|z-z^{*}\left|\right)\pi \left(z,z^{*}\right)\;, \end{array}$$

where **q**_0_ is chosen as the connection probability for long-range and local circuits match up at |*z* − z* | = 0.15, *π*(*z*,z*) = 1 if and only if *z* and z* has the same orientation preference (and equals 0 otherwise). The long-range 6E-to-6I connection function, *p*_*I*_(*d*), is defined analogously. We have assumed also that *p*_*E*_(1.6) = *p*_*I*_(1.6) = 0. See Table E of S1 Text for the table of values of *p*_*E*_(*d*) and *p*_*I*_(*d*) at different interpolation points. Functions *p*_*E*_(*d*) and *p*_*I*_(*d*) are piecewise linear between those interpolation points.

It remains to give the coupling weight from the ambient drive, denoted by *Amb*_4*E*_,*Amb*_4*I*_, *Amb*_6*E*_, and *Amb*_6*I*_. We assume the ambient drive to each type of neuron group is a constant that is small comparing with other terms. Parameters of ambient drives can be found in Table A-D of S1 Text.

#### 4.1.3 The linear equation

In our linear model, the output of each group of neurons is determined by a linear equation. The idea is that the circuit reaches a steady state quickly. At a steady state, the total effect of the net current received by a group of neurons from the entire circuit should be proportional to its output. That is to say, for group *n*, we have

$$\begin{array}{ccc} & & \sum (\text{E}\;\text{weight}\times \text{E}\;\text{current})+\text{Amb}-\sum (\text{I}\;\text{weight}\times \text{I}\;\text{current})\\ & + & \sum (\text{LGN}\;\text{weight}\times \text{LGN}\;\text{current})=c\text{Output}(n)\;, \end{array}$$ (6)

where the current is equal to the output of a presynaptic neuron group multiplied by the corresponding connection probability, *Output*(*n*) is the output of group *n*, *Amb* is the current from ambient drive, and *c* is a constant. Here the three summations are taken over all E groups that project to *n*, all I groups that project to *n*, and all LGN cells that project to *n*, respectively. We can easily absorb *c* into coupling weights by rescaling parameters. Hence from now on we set *c* = 1. Note that Eq (6) is just a more precise form of Eq (1).

Let *z* ∈ Ω be the coordinate of a lattice point in Fig 2C. Denote *Output*_*Q*_(*z*) as the output of a type-*Q* neuron group at location *z*, where *Q* ∈ {4*E*, 4*I*, 6*E*, 6*I*}. Then *Output*_*Q*_ satisfies the linear equation given by Eq (6). Recall that for *Q*_1_,*Q*_2_ ∈ {*LGN*, 4*E*, 4*I*, 6*E*, 6*I*}, the coupling weight from a *Q*_1_-neuron group at *z* to a *Q*_2_-neuron group at z* is denoted by $q_{Q_{1}Q_{2}}\left(z,z^{*}\right)$. And the input of the LGN cell at location *z* is *LGN*(*z*). Take a 4E neuron group *n* at location z* as an example. Eq (6) for a 4E neuron group located at z* ∈ Ω becomes

$$\begin{array}{ccc} & & \sum_{z\in \Omega }q_{LGN4E}\left(z,z^{*}\right)\omega \left(z^{*}\right)\text{LGN}\left(z\right)+\sum_{z\in \Omega }q_{4E4E}\left(z,z^{*}\right){\text{Output}}_{4E}\left(z\right)\\ & + & \sum_{z\in \Omega }q_{6E4E}\left(z,z^{*}\right){\text{Output}}_{6E}\left(z\right)-\sum_{z\in \Omega }q_{4I4E}\left(z,z^{*}\right){\text{Output}}_{4I}\left(z\right)+{\text{Amb}}_{4E}={\text{Output}}_{4E}\left(z^{*}\right)\;, \end{array}$$ (7)

where *ω*(z*) is the orientation value of the neuron group at z*. It take values 1, 0.4, 0.4, and 0.1 if the neuron group at z* prefers orientation 90°, 45°, 135°, and 0°, respectively.

Similarly, we have equations

$$\begin{array}{ccc} & & \sum_{z\in \Omega }q_{LGN4I}\left(z,z^{*}\right)\text{LGN}\left(z\right)+\sum_{z\in \Omega }q_{4E4I}\left(z,z^{*}\right){\text{Output}}_{4E}\left(z\right)\\ & + & \sum_{z\in \Omega }q_{6E4I}\left(z,z^{*}\right){\text{Output}}_{6E}\left(z\right)-\sum_{z\in \Omega }q_{4I4I}\left(z,z^{*}\right){\text{Output}}_{4I}\left(z\right)+{\text{Amb}}_{4I}={\text{Output}}_{4I}\left(z^{*}\right) \end{array}$$ (8)

for the 4I group at z*. Note that the LGN current to 4I neuron groups has no orientation preference.

Equations for 6E and 6I neuron groups at z* are given analogously by

$$\begin{array}{ccc} & & \sum_{z\in \Omega }q_{LGN6E}\left(z,z^{*}\right)\omega \left(z^{*}\right)\text{LGN}\left(z\right)+\sum_{z\in \Omega }q_{4E6E}\left(z,z^{*}\right){\text{Output}}_{4E}\left(z\right)\\ & + & \sum_{z\in \Omega }q_{6E6E}\left(z,z^{*}\right){\text{Output}}_{6E}\left(z\right)-\sum_{z\in \Omega }q_{6I6E}\left(z,z^{*}\right){\text{Output}}_{6I}\left(z\right)+{\text{Amb}}_{6E}={\text{Output}}_{6E}\left(z^{*}\right)\;, \end{array}$$ (9)

and

$$\begin{array}{ccc} & & \sum_{z\in \Omega }q_{LGN6I}\left(z,z^{*}\right)\text{LGN}\left(z\right)+\sum_{z\in \Omega }q_{4E6I}\left(z,z^{*}\right){\text{Output}}_{4E}\left(z\right)\\ & + & \sum_{z\in \Omega }q_{6E6I}\left(z,z^{*}\right){\text{Output}}_{6E}\left(z\right)-\sum_{z\in \Omega }q_{6I6I}\left(z,z^{*}\right){\text{Output}}_{6I}\left(z\right)+{\text{Amb}}_{6I}={\text{Output}}_{6I}\left(z^{*}\right)\;. \end{array}$$ (10)

Given a grating with specified orientation and radius *R*, quantity *LGN*(*z*) is pre-calculated when modeling the LGN input. Combining Eqs (7), (8), (9) and (10) for all z* ∈ Ω, we have a well-defined linear system with 17 × 17 × 7 × 7 × 4 = 56644 variables. We denote this linear equation by

$$\begin{array}{c} H\Theta =b\;, \end{array}$$

where entries of $\Theta \in R^{56644}$ consist of all z* ∈ Ω, $H\in R^{56644\times 56644}$ and $b\in R^{56644}$ are constant matrix and vectors respectively. Solving this linear system gives the E- and I- output profile in L4 and L6. To get the size tuning curve, we solve the linear system for 32 different radii 0.05, 0.1, …, 1.6, and measure the output of the center neuron group in each solution. These outputs are then subtracted by the background output when turning off the LGN (letting *LGN*(*z*) = 0, and plotted versus the grating radii). This gives size tuning curves demonstrated in **Results**.

### 4.2 Nonlinear model

The nonlinearity in this model means some coupling weights are firing rate dependent. In the first nonlinear model, we assume an increase of coupling weights for 6E-to-4I and 6E-to-6E connections when the firing rates of a presynaptic 6E group exceeds a certain level. We also assume an increase of 4E-to-4E coupling weight with the high firing rate of a presynaptic 4E group. The increase is capped by a certain upper bound to avoid undesired effects. Take the 6E-to-4I connection as an example. The coupling weight **q**_6*E*4*I*_(*z*,z*) is replaced by

$$\begin{array}{c} {\tilde{q}}_{6E4I}\left(z,z^{*},\eta \right)=m_{6E4I}\left(\eta \right)q_{6E4I}\left(z,z^{*}\right)\;, \end{array}$$

where *η* is the output of the neuron group at location *z*, and *m*_6*E*4*I*_(*η*) is a multiplier depending on *η*. Then Eq (8) becomes

$$\begin{array}{ccc} & & \sum_{z\in \Omega }q_{LGN4I}\left(z,z^{*}\right)\text{LGN}\left(z\right)+\sum_{z\in \Omega }q_{4E4I}\left(z,z^{*}\right){\text{Output}}_{4E}\left(z\right)\\ & + & \sum_{z\in \Omega }{\tilde{q}}_{6E4I}\left(z,z^{*},{\text{Output}}_{6E}\left(z\right)\right){\text{Output}}_{6E}\left(z\right)-\sum_{z\in \Omega }q_{4I4I}\left(z,z^{*}\right){\text{Output}}_{4I}\left(z\right)+{\text{Amb}}_{4I}\\ & = & {\text{Output}}_{4I}\left(z^{*}\right)\;. \end{array}$$ (11)

Treatments of nonlinear coupling weights for 4E-to-4E and 6E-to-6E connections are analogous. See S1 Text for full details of the nonlinear model. This makes Eqs (7), (8), (9), and (10) a nonlinear system of the form

$$\begin{array}{c} H(\Theta )\Theta =b\;, \end{array}$$

where entries of $\Theta \in R^{56644}$ consist of all z* ∈ Ω, **H**(Θ) is a 56644 × 56644 matrix that depends on **Θ**, and $b\in R^{56644}$ is a constant vector. The solution to this nonlinear system is the output of the nonlinear model. Size tuning curves in **Results** are obtained in the same way as that of the linear model. Note that outputs of the center neuron group are subtracted by the background output, and plotted with radii 0.05 − 1.6.

All parameters are identical to those in the linear model, except two long-range weight functions *p*_*E*_(*d*) and *p*_*I*_(*d*) are slightly modified to address the change caused by the nonlinearity. See S1 Text for the details. Details of three multipliers *m*_6*E*4*I*_(*η*), *m*_4*E*4*E*_(*η*), and *m*_6*E*6*E*_(*η*) are also given in Table F of S1 Text.

## Supporting information

S1 Text Supplementary information for Unraveling the mechanisms for surround suppression in early visual processing. (PDF)
