# Supplementary material for: Unraveling the mechanisms of surround suppression in early visual processing
Source: PLoS Comput Biol. 2021 Apr 22;17(4):e1008916. doi: 10.1371/journal.pcbi.1008916 (PMC8104395; doi:10.1371/journal.pcbi.1008916)
Supplement: S1 Text — (PDF) [file pcbi.1008916.s001.pdf]

Supplementary information: Unraveling the mechanisms of surround suppression in early visual processing

Yao Li[1], Lai-Sang Young[2, 3*],

1Department of Mathematics and Statistics, University of Massachusetts Amherst, Amherst, Massachusetts
2 Courant Institute of Mathematical Sciences, New York University, New York, New York
3 Institute for Advanced Study, Princeton, New Jersey

* lsy@cims.nyu.edu

Equations and parameters

Linear model

Let

$$\Omega = \left\{ z = (x, y) \,|\, x = \frac{h}{2} + mh, y = \frac{h}{2} + nh, 0 \le m, n \le 118 \right\}$$

for $h = 1/28$ (deg). The linear model is given by the following linear system

$$\begin{aligned} &\sum_{z\in\Omega} \mathbf{q}_{LGN4E}(z, z^*)\omega(z^*)\text{LGN}(z) + \sum_{z\in\Omega} \mathbf{q}_{4E4E}(z, z^*)\text{Output}_{4E}(z) \\ + &\sum_{z\in\Omega} \mathbf{q}_{6E4E}(z, z^*)\text{Output}_{6E}(z) - \sum_{z\in\Omega} \mathbf{q}_{4I4E}(z, z^*)\text{Output}_{4I}(z) + \text{Amb}_{4E} = \text{Output}_{4E}(z^*)\,, \end{aligned} \tag{1}$$

$$\begin{aligned} &\sum_{z\in\Omega} \mathbf{q}_{LGN4I}(z, z^*)\text{LGN}(z) + \sum_{z\in\Omega} \mathbf{q}_{4E4I}(z, z^*)\text{Output}_{4E}(z) \\ + &\sum_{z\in\Omega} \mathbf{q}_{6E4I}(z, z^*)\text{Output}_{6E}(z) - \sum_{z\in\Omega} \mathbf{q}_{4I4I}(z, z^*)\text{Output}_{4I}(z) + \text{Amb}_{4I} = \text{Output}_{4I}(z^*) \end{aligned} \tag{2}$$

$$\begin{aligned} &\sum_{z\in\Omega} \mathbf{q}_{LGN6E}(z, z^*)\omega(z^*)\text{LGN}(z) + \sum_{z\in\Omega} \mathbf{q}_{4E6E}(z, z^*)\text{Output}_{4E}(z) \\ + &\sum_{z\in\Omega} \mathbf{q}_{6E6E}(z, z^*)\text{Output}_{6E}(z) - \sum_{z\in\Omega} \mathbf{q}_{6I6E}(z, z^*)\text{Output}_{6I}(z) + \text{Amb}_{6E} = \text{Output}_{6E}(z^*)\,, \end{aligned} \tag{3}$$

$$\begin{aligned} &\sum_{z\in\Omega} \mathbf{q}_{LGN6I}(z, z^*)\text{LGN}(z) + \sum_{z\in\Omega} \mathbf{q}_{4E6I}(z, z^*)\text{Output}_{4E}(z) \\ + &\sum_{z\in\Omega} \mathbf{q}_{6E6I}(z, z^*)\text{Output}_{6E}(z) - \sum_{z\in\Omega} \mathbf{q}_{6I6I}(z, z^*)\text{Output}_{6I}(z) + \text{Amb}_{6I} = \text{Output}_{6I}(z^*)\,, \end{aligned} \tag{4}$$

for $z^* \in \Omega$. For $Q_1, Q_2 \in \{4E, 4I, 6E, 6I, LGN\}$, the coupling weight $\mathbf{q}_{Q_1Q_2}$ sis given by

$$\mathbf{q}_{Q_1Q_2}(z, z^*) = \frac{h^2 W_{Q_1Q_2}}{2\pi\sigma_{Q_1Q_2}} e^{-|z-z^*|^2/2\sigma^2_{Q_1Q_2}} \mathbf{1}_{\{|z-z^*| \le R_{Q_1Q_2}\}}\,,$$

where W, σ, R means the coupling weight, the standard deviation of the Gaussian kernel, and the radius of the connection footprint, respectively. The orientation function $\omega(z^*) = 1, 0.4, 0.4$, and 0.1 if the neuron group at z^* prefers orientation $90°, 45°, 135°$, and $0°$, respectively.

There are also the long-range connections 6E-6E and 6E-6I. When $|z - z^*| > R_{6E6E}$ (resp. $|z - z^*| > R_{6E6I}$), $\mathbf{q}_{6E6E}(z, z^*)$ (resp. $\mathbf{q}_{6E6I}(z, z^*)$) is given by

$$\mathbf{q}_{6E6E}(z, z^*) = \frac{h^2 W_{6E6E}}{2\pi\sigma_{6E6E}} e^{-R_{6E6E}^2/2\sigma_{6E6E}^2} p_E(|z - z^*|)\pi(z, z^*)$$

(resp.

$$\mathbf{q}_{6E6I}(z, z^*) = \frac{h^2 W_{6E6I}}{2\pi\sigma_{6E6I}} e^{-R_{6E6I}^2/2\sigma_{6E6I}^2} p_I(|z - z^*|)\pi(z, z^*)\,.$$

)

Function $\pi(z, z^*) = 1$ if neuron groups at z and z^* have the same orientation preference, and $\pi(z, z^*) = 0$ otherwise.

Below are tables of parameters. Table A, B, C, and D gives connection weights, standard deviations of Gaussian kernels, and radii of connection footprints. Table F gives values of $p_E(d)$ and $p_I(d)$ at interpolation points. Finally, strengths of ambient drive are $\text{Amb}_{4E} = 0.015$, $\text{Amb}_{4I} = 0.03$, $\text{Amb}_{6E} = 0.012$, and $\text{Amb}_{6I} = 0.03$.

Parameter	W_{4E4E}	σ_{4E4E}	R_{4E4E}	W_{6E4E}	σ_{6E4E}	R_{6E4E}
Value	1.44	0.077	0.154	Control	0.07	0.14
Parameter	W_{LGN4E}	σ_{LGN4E}	R_{LGN4E}	W_{4I4E}	σ_{4I4E}	R_{4I4E}
Value	0.3	0.2	0.2	0.9	0.055	0.0825

Table A. Parameters for coupling weights and connectivities to L4 E groups.

Parameter	W_{4E4I}	σ_{4E4I}	R_{4E4I}	W_{6E4I}	σ_{6E4I}	R_{6E4I}
Value	0.95	0.077	0.154	0.81	0.07	0.14
Parameter	W_{LGN4I}	σ_{LGN4I}	R_{LGN4I}	W_{4I4I}	σ_{4I4I}	R_{4I4I}
Value	0.25	0.2	0.2	0.7	0.055	0.0825

Table B. Parameters for coupling weights and connectivities to L4 I groups.

Parameter	W_{4E6E}	σ_{4E6E}	R_{4E6E}	W_{6E6E}	σ_{6E6E}	R_{6E6E}
Value	0.42	0.06	0.06	0.943	0.0748	0.15
Parameter	W_{LGN6E}	σ_{LGN6E}	R_{LGN6E}	W_{6I6E}	σ_{6I6E}	R_{6I6E}
Value	0.12	0.2	0.2	0.7	0.05	0.1

Table C. Parameters for coupling weights and connectivities to L6 E groups.

Parameter	W_{4E6I}	σ_{4E6I}	R_{4E6I}	W_{6E6I}	σ_{6E6I}	R_{6E6I}
Value	0.75	0.06	0.06	0.96	0.075	0.15
Parameter	W_{LGN6I}	σ_{LGN6I}	R_{LGN6I}	W_{6I6I}	σ_{6I6I}	R_{6I6I}
Value	0.12	0.2	0.2	0.7	0.05	0.1

Table D. Parameters for coupling weights and connectivities to L6 I groups.

Distances d	0.15	0.25	0.35	0.45	0.6	0.8	1.0	1.2	1.6
$p_E(d)$	1.0	0.95	0.9	0.82	0.21	0.04	0.025	0.02	0
$p_I(d)$	1.0	0.52	0.41	0.38	0.355	0.355	0.29	0.015	0

Table E. Values of $p_E(d)$ and $p_I(d)$ at interpolation points.

Nonlinear model

Let Ω be the same set as before. The nonlinear model is given by the following linear system

$$\begin{aligned} & \sum_{z\in\Omega} \mathbf{q}_{LGN4E}(z,z^*)\omega(z^*)\mathrm{LGN}(z) + \sum_{z\in\Omega} \tilde{\mathbf{q}}_{4E4E}(z,z^*,\mathrm{Output}_{4E}(z))\mathrm{Output}_{4E}(z) \\ + & \sum_{z\in\Omega} \mathbf{q}_{6E4E}(z,z^*)\mathrm{Output}_{6E}(z) - \sum_{z\in\Omega} \mathbf{q}_{4I4E}(z,z^*)\mathrm{Output}_{4I}(z) + \mathrm{Amb}_{4E} = \mathrm{Output}_{4E}(z^*)\,, \end{aligned} \tag{5}$$

$$\begin{aligned} & \sum_{z\in\Omega} \mathbf{q}_{LGN4I}(z,z^*)\mathrm{LGN}(z) + \sum_{z\in\Omega} \mathbf{q}_{4E4I}(z,z^*)\mathrm{Output}_{4E}(z) \\ + & \sum_{z\in\Omega} \tilde{\mathbf{q}}_{6E4I}(z,z^*,\mathrm{Output}_{6E}(z))\mathrm{Output}_{6E}(z) - \sum_{z\in\Omega} \mathbf{q}_{4I4I}(z,z^*)\mathrm{Output}_{4I}(z) + \mathrm{Amb}_{4I} \\ & = \mathrm{Output}_{4I}(z^*) \end{aligned} \tag{6}$$

$$\begin{aligned} & \sum_{z\in\Omega} \mathbf{q}_{LGN6E}(z,z^*)\omega(z^*)\mathrm{LGN}(z) + \sum_{z\in\Omega} \mathbf{q}_{4E6E}(z,z^*)\mathrm{Output}_{4E}(z) \\ + & \sum_{z\in\Omega} \tilde{\mathbf{q}}_{6E6E}(z,z^*,\mathrm{Output}_{6E}(z))\mathrm{Output}_{6E}(z) - \sum_{z\in\Omega} \mathbf{q}_{6I6E}(z,z^*)\mathrm{Output}_{6I}(z) + \mathrm{Amb}_{6E} \\ & = \mathrm{Output}_{6E}(z^*)\,, \end{aligned} \tag{7}$$

and

$$\begin{aligned} & \sum_{z\in\Omega} \mathbf{q}_{LGN6I}(z,z^*)\mathrm{LGN}(z) + \sum_{z\in\Omega} \mathbf{q}_{4E6I}(z,z^*)\mathrm{Output}_{4E}(z) \\ + & \sum_{z\in\Omega} \mathbf{q}_{6E6I}(z,z^*)\mathrm{Output}_{6E}(z) - \sum_{z\in\Omega} \mathbf{q}_{6I6I}(z,z^*)\mathrm{Output}_{6I}(z) + \mathrm{Amb}_{6I} = \mathrm{Output}_{6I}(z^*)\,. \end{aligned} \tag{8}$$

For $Q_1, Q_2 \in \{4E, 4I, 6E, 6I, LGN\}$, all linear coupling weights $\mathbf{q}_{Q_1Q_2}(z,z^*)$ are same as those in the linear model. The orientation function $\omega(z^*)$ is also same as in the linear model. The nonlinear coupling weights satisfies

$$\begin{aligned} \tilde{\mathbf{q}}_{4E4E}(z,z^*,\eta) &= m_{4E4E}(\eta)\mathbf{q}_{4E4E}(z,z^*)\,, \\ \tilde{\mathbf{q}}_{6E4I}(z,z^*,\eta) &= m_{6E4I}(\eta)\mathbf{q}_{6E4I}(z,z^*)\,, \\ \tilde{\mathbf{q}}_{6E6E}(z,z^*,\eta) &= m_{6E6E}(\eta)\mathbf{q}_{6E6E}(z,z^*)\,. \end{aligned}$$

The three multipliers $m_{4E4E}(\eta), m_{6E4I}(\eta)$, and $m_{6E6E}(\eta)$ are defined as

$$m_{6E4I}(\eta) = \begin{cases} 1 & \text{if} \quad \eta < 0.19 \\ 1 + 5(\eta - 0.19)/W_{6E4I} & \text{if} \quad 0.19 < \eta \le 0.22 \\ 1 + 0.15/W_{6E4I} & \text{if} \quad \eta > 0.22\,, \end{cases}$$

$$m_{4E4E}(\eta) = \begin{cases} 1 & \text{if} \quad \eta < 0.29 \\ 1 + 0.1(\eta - 0.29)/W_{4E4E} & \text{if} \quad 0.29 < \eta \le 0.39 \\ 1 + 0.01/W_{4E4E} & \text{if} \quad \eta > 0.39\,, \end{cases}$$

and

$$m_{6E6E}(\eta) = \begin{cases} 1 & \text{if} \quad \eta < 0.19 \\ 1 + 0.6(\eta - 0.2)/W_{6E6E} & \text{if} \quad 0.19 < \eta \le 0.21 \\ 1 + 0.012/W_{6E6E} & \text{if} \quad \eta > 0.21 \end{cases}$$

respectively. For $Q_1, Q_2 \in \{LGN, 4E, 4I, 6E, 6I\}$, the coupling weight $W_{Q_1Q_2}$, the standard deviation of the Gaussian kernel $\sigma_{Q_1Q_2}$, and the radius of the footprint $R_{Q_1Q_2}$ are all given by Table A, B, C, and D. The control parameter W_{6E4E} equals 0.48 in the nonlinear model. Two long-range weight functions are slightly adjusted to address the nonlinearity. Values of $p_E(d)$ and $p_I(d)$ at interpolation points are given in the following table.

Distances d	0.15	0.25	0.35	0.45	0.6	0.8	1.0	1.2	1.6
$p_E(d)$	1.0	0.95	0.9	0.82	0.21	0.04	0.023	0.0184	0
$p_I(d)$	1.0	0.52	0.41	0.38	0.36	0.35	0.275	0.0145	0

Table F. Values of $p_E(d)$ and $p_I(d)$ at interpolation points.

Computation methods

Linear solver

Eq (1), Eq (2), Eq (3), and (4) give a linear system with 56644 variables, denoted by

$$\mathbf{H}\boldsymbol{\Theta} = \mathbf{b}\,, \tag{9}$$

where $\mathbf{H}$ is a 56644×56644 matrix, $\mathbf{b}$ is a vector in $\mathbb{R}^{56644}$, and $\boldsymbol{\Theta}$ is the solution that consists of all outputs $\text{Output}_{4E}(z), \text{Output}_{4I}(z), \text{Output}_{6E}(z)$, and $\text{Output}_{6I}(z)$, where z goes through all lattice points. Solving this matrix equation is not easy. Without optimizing the algorithm, our test (in C++) shows that generating matrix $\mathbf{H}$ (with grating radius 0.4) and solving Eq (9) by LU factorization would take 4866.34s total. Note that this is only the cost of solving the output profile for one grating radius. In other words, it takes nearly two days to produce a tuning curve, which needs firing rates for 32 different grating radii and the background rate when LGN is turned off. Computational time like this will make any parameter tunings impossible.

To optimize the computation, the first observation is that the matrix $\mathbf{H}$ in Eq (9) is sparse. A neuron usually only connects to several hundreds postsynaptic neuron groups. The only exception is the L6 excitatory population, which has long range connections to neuron groups with the same orientation preference. Hence we use the iterative solver *BiCGSTAB* for sparse matrices provided in *Eigen* C++ library. The *Eigen* library further calls a basic linear algebra subroutine (BLAS), which is the *OpenBLAS* package in this study. In addition, generating matrix $\mathbf{H}$ is also computationally expensive. But this process can be easily parallelized because all matrix entries can be computed independently. We have also implemented other compiler optimizations to accelerate the computation. A performance test shows that the average time of five runs of the linear model (with grating radius 0.4) is 26.09s, in which the error tolerance is set to be 10^{-6}. This is about 200 times faster than using serial matrix generation and LU linear equation solver. In our computation, the matrix generation and the linear solver together usually take $25 - 35$ seconds, depending on the radius of the grating.

Nonlinear equation

The nonlinear model Eq (5), Eq (6), (7), and (8) gives a nonlinear equation

$$\mathbf{H}(\boldsymbol{\Theta})\boldsymbol{\Theta} = \mathbf{b}\,, \tag{10}$$

where $\mathbf{\Theta}$ and $\mathbf{b}$ are same as in the linear model, and $\mathbf{H}(\Theta)$ is a firing-rate dependent matrix. The dependency of firing rate comes from firing rate dependent coupling weights (4E-to-4E, 6E-to-6E, 6E-to-4I for nonlinear model 1, 4E-to-4E and 6I-to-4E for nonlinear model 2). Solving Eq (10) for both nonlinear models imposes a greater computational challenge. A very heuristic approach is to solve Eq (10) by fixed point iterations. One can start from $\mathbf{\Theta}_0 = \mathbf{0}$ and solve a series of equations

$$\mathbf{\Theta}_{n+1} = \mathbf{H}(\mathbf{\Theta}_n)^{-1}\mathbf{b}$$

to get a sequence $\mathbf{\Theta}_0, \mathbf{\Theta}_1, \mathbf{\Theta}_2, \cdots$. If it converges, the limit point solves Eq (10). However, our numerical simulation shows that the fixed point iteration has poor performance. When it converges, the speed of convergence is often very slow. And it may not even converge when Eq (10) actually has a solution.

To improve the nonlinear solver, an important observation is that Eq (10) can be rewritten as

$$\mathbf{\Theta} - \mathbf{H}(\mathbf{\Theta})^{-1}\mathbf{b} = \mathbf{0}\,. \tag{11}$$

When $\mathbf{\Theta} = \mathbf{0}$, the Jacobian matrix of Eq (11) is the identity matrix because the nonlinearity has not been activated. This is a very important step, because almost all quasi-Newton methods require an approximate Jacobian matrix to start. And the performance is usually not satisfactory if the initial guess of the Jacobian matrix is wrong.

Then we use Broyden's method to solve Eq (11), which is a quasi-Newton method with update

$$\mathbf{\Theta}_{n+1} = \mathbf{\Theta}_n - \mathbf{J}_n^{-1}\left(\mathbf{\Theta}_n - \mathbf{H}(\mathbf{\Theta}_n)^{-1}\mathbf{b}\right)\,,$$

where $\mathbf{J}_n$ is an approximated Jacobian matrix that is updated after each step by the Sherman-Morrison formula. We use the initial guess $\mathbf{\Theta}_0 = \mathbf{0}$ and the initial Jacobian matrix $\mathbf{J}_0 = \mathbf{Id}_{56644}$. When evaluating $\mathbf{\Theta}_n - \mathbf{H}(\mathbf{\Theta}_n)^{-1}\mathbf{b}$, the same iterative solver for the linear model (9) is used to compute $\mathbf{H}(\mathbf{\Theta}_n)^{-1}\mathbf{b}$. Our implementation follows Algorithm 7.3.1 in [1]. In fact, the nonlinearity is only a small modification to the linear system. Hence the solution to the linear system is not very far away from that of the linear system. In practice, the Broyden's method converges fast, especially if the nonlinearity does not change the solution very much. Usually the Broyden's method needs only 3-10 iterations to solve Eq (10) with an error tolerance 10^{-6}.

Parameter tuning

It remains to discuss the technique of our parameter tuning. The linear model has 80 parameters. The nonlinear model has 89 parameters. Hence a parameter sweep clearly does not work. For the linear model, one needs to test $2^{80} \approx 1.21 \times 10^{24}$ parameter sets even if only testing two possible values of each parameter. In addition, most randomly chosen parameter set do not produce a meaningful tuning curve. Instead, we first decide a rough range of parameters based on the anatomy of the visual cortex and experimental results. Then we run the linear model for several different parameter sets to obtain a rough parameter range. It takes only about 2-3 minutes to see the result of the linear model, because one only needs to compute for several grating radii to see values and locations of peaks of tuning curves. This allows us to further adjust parameters.

In addition to manual parameter fitting, we compute partial derivatives with respect to all parameters with respect to the following eight quantities: (1) location of the 4E peak, (2) value of the 4E peak, (3) location of the 6E peak, (4) value of the 6E peak, (6) surround 4E firing rate, (6) surround 6E firing rate, (7) suppression index of 4E, and (8) suppression index of 6E. The partial derivative is computed by taking a finite difference with respect to each corresponding parameter. These derivatives give us an additional

guidance to tune parameters. For example, we will know which parameters are responsible for a higher 4E peak, or a lower 4E surround rate. Then we can move parameters slightly to improve the result. After several rounds, we can obtain a suitable tuning curve for the linear model.

Parameters for the nonlinear model are fitted after having results for the linear model, because computing a nonlinear model is usually 3-5 times slower. In fact it takes us several hours to compute partial derivatives with respect to all parameters for a nonlinear model. The threshold of activating nonlinearity is determined by the linear model. For example, if we want to activate 6I-to-4E connections to suppress the firing rate of 4E groups when the grating is larger than 0.4, we let the center 6I firing rate of the linear model at radius 0.4 be the threshold in function $m_{6I4E}(\eta)$. Then the only remaining terms are slopes and upper bounds of nonlinear multipliers $m_{4E4E}(\eta)$, $m_{6E6E}(\eta)$ etc. This can be done quickly by several manual adjustments after knowing all partial derivatives.

References

1. Kelley CT. Iterative methods for linear and nonlinear equations. vol. 16. Siam; 1995.
